# Ground-State Energy Estimation on Current Quantum Hardware through the Variational Quantum Eigensolver: A Practical Study

**DOI:** 10.1021/acs.jctc.4c01657

**Published:** 2025-06-29

**Authors:** Nacer Eddine Belaloui, Abdellah Tounsi, Abdelmouheymen Rabah Khamadja, Mohamed Messaoud Louamri, Achour Benslama, David E. Bernal Neira, Mohamed Taha Rouabah

**Affiliations:** † Constantine Quantum Technologies, Frères Mentouri University Constantine 1, Ain El Bey Road, Constantine 25017, Algeria; ‡ Laboratoire de Physique Mathématique et Subatomique, Frères Mentouri University Constantine 1, Ain El Bey Road, Constantine 25017, Algeria; § Theoretical Physics Laboratory, University of Science and Technology Houari Boumediene, BP 32 Bab Ezzouar, Algiers 16111, Algeria; ∥ Davidson School of Chemical Engineering, Purdue University, 480 Stadium Road, West Lafayette, Indiana 47907, United States

## Abstract

We investigate the variational quantum eigensolver (VQE) for estimating the ground-state energy of the BeH_2_ molecule, emphasizing practical implementation and performance on current quantum hardware. Our research presents a comparative study of HEA and UCCSD ansätze on noiseless and noisy simulations and implements VQE on recent IBM quantum computer noise models and a real quantum computer, IBM Fez, providing a fully functional code employing Qiskit 1.2. Our experiments confirm UCCSD’s reliability in ideal conditions, while the HEA demonstrates greater robustness to hardware noise, achieving chemical accuracy on state-vector simulation (SVS). The results reveal that achieving ground-state energy within chemical accuracy is feasible without error mitigation during VQE convergence. We demonstrate that current quantum devices effectively optimize circuit parameters despite misestimating simulated energies. The SVS-evaluated energies provide a more accurate representation of the solution quality compared to QPU-estimated energy values, indicating that VQE converges to the correct ground state despite quantum noise. Our study also applies noise mitigation as a postprocessing technique, using zero-noise extrapolation (ZNE) on a real quantum computer. The detailed methodologies presented in this study, including Hamiltonian construction and Fermionic-to-qubit transformations, facilitate flexible adaptation of the VQE approach for various algorithm variants and across different levels of algorithmic implementation.

## Graphical abstract

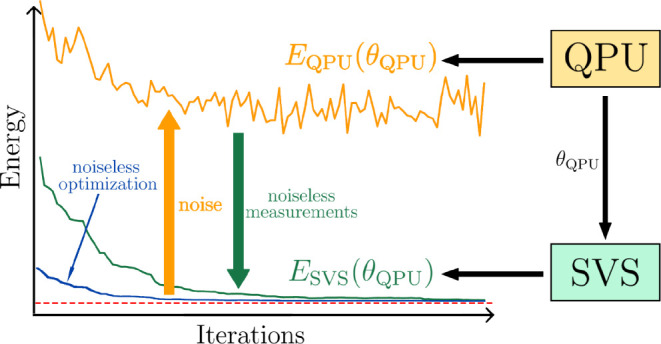

## 1 Introduction

Accurately modeling the behavior of molecules at the quantum level provides invaluable insight into chemical properties and processes that are essential to a variety of modern industries. Quantum chemistry^1–3^ which encapsulates the study of molecules and their properties within quantum mechanics, primarily focuses on the computation of electronic structure properties and their contributions to chemical reactions at the atomic level. In this field, electrons and nuclei are ruled by the Schrödinger equation constructed with the molecular Hamiltonian, and their interactions are described by its solution function, often called the wave function. Quantum chemistry’s ability to offer fundamental insights into molecular properties, electronic structures, and chemical reactions makes it a crucial tool in various scientific domains, including medicinal chemistry and drug design,^4^ materials chemistry,^5^ computational biology,^6^ and environmental chemistry.^7^ For these applications, solving problems related to electronic structure, chemical bonding analysis, molecular ground-state energy, molecular dynamics, and reaction energy are essential.^1–3^ In particular, accurate estimation of the ground-state energy in quantum chemistry is crucial because it provides a fundamental understanding of the most stable configuration of a molecular system. The ground-state energy is the lowest possible energy that a system can occupy, and it directly influences the physical and chemical properties of molecules. Precise knowledge of this energy allows us to predict molecular behavior, design and optimize new materials, control chemical reactions, and develop pharmaceuticals.

Computational quantum chemistry^8–10^ has made significant progress in the past century, with *ab initio* methods^1,11–13^ such as the Coupled-Cluster^12,14^ and Møller–Plesset^2,15,16^ methods, but also semiempirical,^1,12^ Monte Carlo,^17,18^ Density Matrix Renormalization Group (DMRG),^19,20^ Density Functional Theory (DFT),^12,21–23^ and machine learning^24^ methods. These approaches utilize systematic approximations to achieve a targeted accuracy based on available resources. Although, despite their success, these methods have limitations and challenges. From an *ab initio* point of view, to reach a sufficient accuracy in chemical computations, sufficiently large basis sets and Full Configuration Interaction (FCI) wave functions are needed.^12,25^ However, a large basis set requires extensive computational resources,^25^ whereas FCI calculations imply a combinatorial complexity that results in a prohibitive computational cost when the number of electrons and the size of the basis set are large. For instance, to the moment of writing this paper, the largest FCI computation has been performed for the C_3_H_8_ molecule within the Slater-type orbital (STO) 3 Gaussian, *STO-3G*, basis set. The computation included 1.3 × 10^12^ configurations and required 256 servers.^26^ Although computational methods like the DFT give useful predictions and consider electron correlations, they are still approximate and expensive methods for addressing large problems while aiming for an optimal accuracy. Moreover, as the size of the molecular system grows, the resources required to simulate it classically increase exponentially. That is, quantum chemistry problems can be computationally demanding, especially for large molecules or complex systems.^24,26,27^ Furthermore, the interaction between electrons poses a significant challenge for accurately predicting molecular properties, especially in systems with strong electron–electron interactions, which give rise to highly entangled electron states. These states quickly become intractable using *classical* computers since they require heavy FCI computations. Consequently, accurately solving the Schrödinger equation requires significant computational resources, and approximations become necessary, with the cost of sacrificing precision, to scale quantum computations on classical devices.

Quantum computers^28,29^ can overcome some challenges that classical computers face in this matter, and in particular the combinatorial growth of the configuration space of such quantum systems and the correlations within that space.^27^ Indeed, quantum computers would require fewer approximations due to their qubits’ ability to be entangled and manifest other quantum behaviors inherently. These behaviors emerge without the need for additional explicit tracking of correlations and probabilities. By achieving the ability to address these challenges within a feasible time frame without relying extensively on approximations, quantum computers will ultimately enable us to make more accurate predictions regarding various properties of quantum systems.^30^

From the inception of the idea of quantum computers^28,29^ through their realizability criteria,^31^ to current quantum computers^32–34^ numerous algorithms were born in the pursuit of quantum supremacy.^30,32^ An example of the aforementioned algorithms that can be applied to quantum chemistry is the Quantum Phase Estimation (QPE) algorithm^35,36^ which enables the computation of all energy levels of a given Hamiltonian while having a polynomial complexity^35,37^ and would have been of a major benefit to the field if it were not for its intolerance to faults. Implementation of QPE requires a fault-tolerant quantum computer with long coherence times and good qubit connectivity, which is still a challenge for all but the smallest of problems, such as the hydrogen molecule which is solvable using only two qubits.^38^ Currently, efforts are more oriented toward utilizing the current *Noisy Intermediate-Scale Quantum* (NISQ) computers, i.e., quantum computers comprised of relatively small numbers (and up to a few hundred) of noisy qubits with short coherence times.^39^

Variational Quantum Algorithms (VQAs)^40,41^ have garnered significant attention as promising candidates to achieve quantum advantage with NISQ computers.^39^ VQAs have been developed for a broad spectrum of applications, including the determination of molecular ground states, the simulation of quantum system dynamics^30,42^ the solution of linear systems of equations,^43^ and the solution of discrete optimization problems.^44^ VQAs share a unified framework in which tasks are encoded into parametrized cost functions that are evaluated using a quantum computer. A classical optimizer subsequently trains the parameters within the VQA. The inherent adaptability of VQAs makes them particularly well-suited to address the limitations posed by near-term quantum computing technologies. In this pursuit, Peruzzo et al.^45^ introduced the Variational Quantum Eigensolver (VQE), a hybrid quantum-classical algorithm that approximates the lowest eigenvalue of a given Hamiltonian. The performance of the VQE algorithm depends on the quality of many components, such as the choice of the ansatz quantum circuit^46^ and the classical optimizer.^47,48^ The fundamental difference between QPE and VQE is that the former requires the implementation of *O*(1) quantum circuits with depth *O*(1/ϵ) to achieve an energy accuracy of ϵ. In contrast, the latter requires the implementation of *O*(1/ϵ^2^) quantum circuits with depth *O*(1) at each iteration,^40,41,49^ Nevertheless, the VQE faces significant challenges that limit its current advantages over classical methods for certain applications. One major bottleneck is the high cost of measuring the expectation value of the Hamiltonian, requiring a large number of measurements, especially for complex systems. Although research into efficient operator sampling and parallelization offers potential solutions, these approaches would necessitate a paradigm shift in quantum hardware design. Another limitation lies in the optimization process, which is inherently NP-hard,^50^ with convergence depending on the specific problem’s optimization landscape and the choice of optimizer. Additionally, the presence of barren plateaus in this landscape, where the gradients of the cost function vanish exponentially with system size,^51^ poses severe scaling issues, making optimization intractable for certain parametrizations. Although mitigation strategies, such as identity block initialization and local Hamiltonian encoding, have been proposed, their effectiveness for large-scale systems remains an open question. Similarly, while the VQE shows inherent noise resilience due to its variational nature, error mitigation techniques are often required to achieve accurate results on noisy quantum devices. These methods can significantly increase resource demands, and it is unclear whether this trade-off will be manageable for large-scale applications.

It is worth noting that VQE has undergone various improvements to address both implementation and algorithmic challenges. For instance, Adapt-VQE was introduced to mitigate optimizability issues by employing gradient-free iterative methods.^52^ In a similar vein, the Qubit Coupled Cluster (QCC) method constructs the ground state circuit progressively through a hierarchy of quantum circuit-friendly qubit entanglers.^53^ One other interesting iterative algorithm is the Contracted Quantum Eigensolver (CQE) which follows an iterative construction of the ansatz to converge to the eigenstates of the electronic Hamiltonian^54–56^ and even of mixed Fermion-boson Hamiltonians.^57^ On the other hand, significant efforts have been made to improve the performance of chemically inspired ansätze, such as Unitary Coupled Cluster (UCC), UCC with Singles and Doubles (UCCSD), generalized UCCSD, and k-UpCCGSD, optimizing them for practical quantum simulations.^58^ Additionally, accuracy improvements have been explored through Jastrow-based ansätze,^59^ which integrate correlations in a compact and hardware-efficient manner.

Moreover, many nonvariational alternative eigen-solving methods have been developed such as the Connected Moments Expansion (CME) method based on Horn-Weinstein theorem,^60^ and the Variational Quantum Imaginary Time Evolution (Var-QITE) methods based on McLachlan principle.^102^ In recent times, quantum subspace-based diagonalization methods^61^ appeared as a serious candidate to deliver near-term advantage, one of which is the Quantum Selected Configuration Interaction (QSCI),^62^ a hybrid approach that does not rely on accurate energy estimation. Applications of methods built on QSCI such as Sample-based Quantum Diagonalization (SQD) have already been implemented: simulation of N_2_ triple bond breaking and the active-space electronic structure of [2Fe–2S] and [4Fe–4S] clusters,^63^ accurate simulations of supramolecular interactions,^64^ and simulations of the open-shell methylene CH2 singlet and triplet states.^65^ In spite of the fact that these methods do not directly estimate the energy from the QPU but rather compute it classically using samples obtained from the quantum computer, they still rely on a good approximation for the ground state that can be prepared using the VQE.

Despite its challenges, the VQE has shown success in small-scale implementations and holds promise as one of the earliest practical algorithms for implementation on NISQ devices. However, realizing its full potential requires continuous advancements not only in quantum hardware but also in the theoretical framework, as well as in the efficiency and robustness of the associated software and algorithms. Such progress is essential to ensure that the benefits of approaches like the VQE can be effectively harnessed at the earliest opportunity.

This paper serves as a practical and thorough guide for utilizing the VQE to estimate molecular ground-state energy, specifically focusing on a single molecular geometry. Therefore, we preeminently explore all the steps and components of the VQE and investigate chemically inspired and hardware-efficient ansätze involving a small number of qubits. We specifically benefit from the efficiency of the Simultaneous Perturbation Stochastic Approximation (SPSA) optimizer^66^ to mitigate the effects of noise on the evaluation of our cost function.^47^ By dissecting this process, we aim to provide a foundational understanding of the entire VQE pipeline that can be extended to multiple molecular configurations, ultimately aiding in the exploration of ground and excited states energies and prediction of molecular dynamics.

Furthermore, to examine the influence of noise on VQE performance—an essential step toward understanding and enhancing its resilience to noise, especially as quantum hardware advances—we compare state-vector noiseless simulation results to those obtained from noisy quantum circuit simulations using noise models of three recent IBM quantum computers with different error rates, namely IBM Strasbourg, IBM Torino, and IBM Fez. Additionally, we implement the algorithm on the actual IBM Fez quantum computer, and compare the SVS-evaluated energies quality to the energy values estimated on the quantum hardware to highlight the VQE’s trainability under noisy conditions. For the sake of this study, we use the VQE to estimate the ground state energy of the BeH_2_ molecule at a specific Be–H bond length near its equilibrium geometry,^67^ as illustrated in Figure 1.

**Figure 1:**
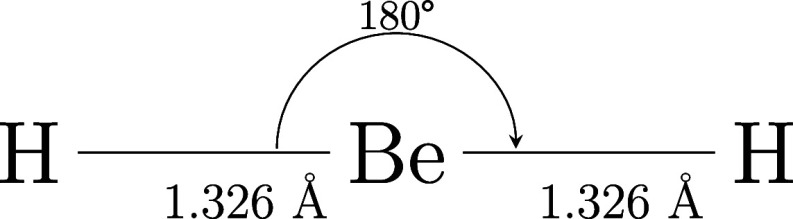
Experimental equilibrium molecular geometry of BeH_2_.

This paper is organized as follows: Section 2 covers the process of building the Hamiltonian, describing the classical subroutines of the VQE,^68^ starting with molecular geometry and molecular orbitals, moving through the second quantization, and concluding with the transformation from Fermionic to Pauli operators. In Section 3, we discuss the VQE, the classical optimization, and the specific ansätze employed in our study. Section 4 presents our experiments and findings, detailing our simulation results, and the implementation of our VQE on a real IBM quantum computer using Qiskit 1.2, the latest version of IBM’s SDK at the time of completing this work. Further quantum chemistry computations and details relevant to building a molecular Hamiltonian, as well as documented codes for the VQE are provided in this paper’s Supporting Information.

## 2 Building the Molecular Hamiltonian

In the context of molecular problems, we study the dynamics of a molecule that is comprised of a number of nuclei and electrons, all of which are interacting with each other through the Coulomb force. The general molecular Hamiltonian will thus take the following form:

1 $$H_{mol}=-\underset{n}{\sum }\frac{\nabla_{n}^{2}}{2M_{n}}-\underset{i}{\sum }\frac{\nabla_{i}^{2}}{2}-\underset{m,i}{\sum }\frac{Z_{m}}{\left|R_{m}-r_{i}\right|}+\underset{m,n>m}{\sum }\frac{Z_{m}Z_{n}}{\left|R_{m}-R_{n}\right|}+\underset{i,j>i}{\sum }\frac{1}{\left|r_{i}-r_{j}\right|}$$

in the atomic units. For clarity, we use *m*, *n* to sum over nuclei, *i*, *j* to sum over electrons, and we use **R** and **r** to represent position vectors of nuclei and electrons, respectively. The first two terms of ([Disp-formula eq1]) are the kinetic energy terms of the nuclei and electrons, respectively, while the last three terms describe (in order) the electron–nucleus interactions, nucleus–nucleus interactions, and electron–electron interactions. This molecular Hamiltonian can be simplified by transforming it into an electronic Hamiltonian, i.e., a problem where we only solve for the dynamics of the electrons. This is achieved using the Born–Oppenheimer approximation^1,69^ on account of the large difference between the masses of an electron and that of a nucleus, resulting in a noticeable difference between the speed and frequency of their motion. This approximation does not hold under the Jahn–Teller effect where the *conical intersection* takes place, and the excited state interacts with the ground state.^70,71^ In this approximation, the nuclei’s kinetic energy term, $\underset{n}{\sum }\frac{\nabla_{n}^{2}}{2M_{n}}$ , tends to zero. In contrast, the nucleus–nucleus repulsion term, $\underset{m,n>m}{\sum }\frac{Z_{m}Z_{n}}{\left|R_{m}-R_{n}\right|}$ , becomes a constant that can be computed classically. After the simplification of the initial Hamiltonian, we obtain the following electronic Hamiltonian:

2 $$H_{el}=-\underset{i}{\sum }\frac{\nabla_{i}^{2}}{2}-\underset{m,i}{\sum }\frac{Z_{i}}{\left|R_{m}-r_{i}\right|}+\underset{i,j>i}{\sum }\frac{1}{\left|r_{i}-r_{j}\right|}$$

which acts on the wave function Ψ­(**x**_1_, **x**_2_, ..., **x**_*N*_), where **x**_*i*_ = (**r**_*i*_, *s*_*i*_) describes the spatial position and the spin of the *i*’th electron. To solve for the ground state of the Hamiltonian, quantum mechanical approaches such as *ab initio* methods^1,12^ semiempirical methods^2,12^ and DFT-based approaches^12,21^ are considered. In this work, we follow the *ab initio* approach, which is based on describing the wave function as a linear combination of Slater determinants of the occupied molecular orbitals. Defining an orthonormal set of molecular orbital allows for the representation of the electronic state as a Fock state, which will be practical for the second quantization of the electronic Hamiltonian in the subsequent section. The molecular orbitals are expanded as a Linear Combination of Atomic Orbitals (LCAO), which in turn are written in a basis set of Gaussian primitives. Details on this expansion are provided in the Supporting Information. Then, the Self-Consistent Field (SCF) method is used to find the values of LCAO coefficients and consequently determines the Hartree–Fock reference state.

Finding an exact solution of the Schrödinger equation within a given basis set is equivalent to solving the Full Configuration Interaction (FCI) functions, where the wave function of a molecule is expressed as a linear combination of all possible Slater determinants that can be constructed from a given set of molecular orbitals. However, for a number of electrons *N* and a number of molecular orbitals *M*, the number of possible occupation configurations increases as (2MN) . Therefore, it is more convenient to use a quantum computer to deal with such factorially growing search space^27^ employing a number of qubits on the scale of *O*(log_2_(*D*)), where *D* is the number of determinants. However, the electronic Hamiltonian in the first quantized form, shown in eq 2, is not suitable to simulate and solve for on a quantum computer. Therefore, we need to transform the Hamiltonian to the second quantized operators’ form, as the latter will require a finite number of qubits and is more easily mapped into quantum gates. The electronic state in the second quantized form will be represented as a Fock state that encodes the occupation state of each molecular spin orbital. Thus, it represents the Slater determinant of the occupied orbitals. The quantum computation advantage lies in the ability to store the coefficients of different Slater determinants in a single quantum register.

### 2.1 The Second Quantization of Electronic Hamiltonian

Since the electronic Hamiltonian involves one- and two-body interaction terms, the second-quantized Hamiltonian can be written under the form:

3 $$H_{el}=\underset{p,q}{\sum }h_{pq}a_{p}^{†}a_{q}+\underset{p,q,r,s}{\sum }h_{pqrs}a_{p}^{†}a_{q}^{†}a_{r}a_{s}$$

with *a* ^†^ and *a* being the electron creation and annihilation operators. The first term thus represents the transitions of single electrons between different orbitals, while the second term corresponds to the simultaneous transitions of electron pairs between different orbitals. The coefficients *h*_*pq*_ and *h*_*pqrs*_ are the one- and two-electron integrals defined as^8,12^

4 $$h_{pq}=\int \psi_{p}^{*}\left(x\right)\left(\frac{-\nabla^{2}}{2}-\underset{i}{\sum }\frac{Z_{i}}{\left|R_{i}-r\right|}\right)\psi_{q}\left(x\right)dx$$

5 $$h_{pqrs}=\int \frac{\psi_{p}^{*}\left(x_{1}\right)\psi_{q}^{*}\left(x_{2}\right)\psi_{r}\left(x_{1}\right)\psi_{s}\left(x_{2}\right)}{\left|r_{1}-r_{2}\right|}dx_{1}dx_{2}$$

where ψ­(**x**) is the molecular spin orbital’s wave function, and **x** encapsulates both the electron’s position and spin, as defined earlier.

For some selected cases, these integrals can be computed analytically or numerically in a reasonable amount of time. This is especially true in the case of the Gaussian expansion of Slater orbitals. Beyond the simpler 1*s* type orbitals (see Supporting Information), the computation of *p* and higher orbital types’ integrals are proven to be efficient using different methods.^3,72^

### 2.2 Fermionic to Pauli Operators Transformation

The creation and annihilation operators, *a* ^†^ and *a*, introduced in the second quantized Hamiltonian ([Disp-formula eq3]) are not native to gate-based quantum computers, the latter operating mainly on qubits with Pauli operators. However, a transition from Fermionic to Pauli operators is challenging because it is necessary to maintain the Fermionic anticommutation relations, while single-qubit Pauli operations can only give rise to the bosonic algebra. Several methods have been developed to address this requirement; the most popular include the Jordan–Wigner,^73^ Parity,^74^ and Bravyi–Kitaev^75^ transformations. Although the Jordan–Wigner transformation is the natural starting point from an analytical point of view, the successor Parity and Bravyi–Kitaev transformations can be more advantageous. The Parity transformation can introduce a $Z_{2}$ symmetry that allows for two-qubit tapering.^76,77^ The Bravyi–Kitaev transformation has the advantage of scaling the weight of Pauli terms, i.e., the number of nontrivial local Pauli operators, logarithmically with the number of qubits instead of linearly. Moreover, different Fermionic mapping methods do not, in general, yield the same number of Pauli terms and can differ in measurement performance. In this work, we use the Parity transformation and qubit tapering since they allow for resource reduction and provide, in this case, lighter Pauli terms that require fewer local measurements, as shown in Table 1.

**Table 1:** In the BeH_2_ Case, All Three Transformations Require the Same Number of Qubits and Produce the Same Number of Terms^a^,^b^

Active orbitals	Active electrons	Mapping	Qubits	Hamiltonian terms	Pauli terms	Average weight
3	2 or 4	Parity	6	91	34	3.12
		Parity (2-qubit tapered)	4	91	28	2.57
		Jordan–Wigner	6	91	34	2.71
		Bravyi–Kitaev	6	91	34	3.24
7	6	Parity	14	1939	666	6.12
		Parity (2-qubit tapered)	12	1939	666	5.69
		Jordan–Wigner	14	1939	666	5.82
		Bravyi–Kitaev	14	1939	666	5.96

a The Parity mapping allows for a two-qubit reduction due to the introduced $Z_{2}$ symmetry due to the conserved number of α and β electrons.

b In addition, the average weight of the Pauli terms is computed for each method, which indicates the average number of local Pauli measurements required for each term.

In the Parity transformation, the Fock state is represented as

6 $$\left|\psi \right\rangle =\left|e_{0}e_{1}\cdot \cdot \cdot e_{k}\right\rangle$$

such that $e_{i}=\left(\overset{i-1}{\underset{j=0}{\sum }}n_{j}\mathrm{mod}2\right)$ , where *n*_*j*_ is the occupation number of the orbital *j* and *e*_*i*_ is the parity of the sum of all occupied orbitals up to the *i*’th, hence the name. Consequently, the ladder operators are given by

7 $$a_{p}=\frac{1}{2}\left(X_{p}Z_{p-1}-iY_{p}\right)X_{p+1}\cdot \cdot \cdot X_{k}$$

with

8 $$\left\{a_{p},a_{q}\right\}=\left\{a_{p}^{†},a_{q}^{†}\right\}=0$$

9 $$\left\{a_{p},a_{q}^{†}\right\}=\delta_{pq}$$

In practice, the α and β spin sector electrons (spin up and down electrons) can be encoded separately in the Fock state. Notice that the last Pauli operator of $a_{p}^{†}a_{q}$ is either *I*_*k*_ or *Z*_*k*_ and hence commutes with *Z*_*k*_. Knowing that the number of electrons in the spin up and down sectors is conserved in the electronic Hamiltonian, it is possible to encode α and β modes in a bipartite set of qubits. This results in a fixed parity that is encoded in the last qubit of each part. Due to this $Z_{2}$ symmetry, it is possible to taper one qubit from each spin sector if the total spin *S* ^2^ is fixed *a priori*.^76,77^ It is worth mentioning that the mapping to Pauli operators is classically efficient since it involves linear relations between ladder and Pauli operators.

In the case of BeH_2_, the required number of qubits and Pauli terms is affected by the amount of approximation introduced by fixing the number of active orbitals and electrons as shown in Table 1. Such a heavy approximation is not in general recommended. Still, it is necessary in the case of small quantum devices that do not have the required number of high-quality qubits for larger Hamiltonians. However, qubit tapering provides a qubit number reduction without introducing approximations by fixing the number of electrons in each of the α and β spin sectors.

## 3 The Variational Quantum Eigensolver

The variational method in quantum mechanics, and by extension, the variational quantum eigensolver, relies on a trial quantum state to be parametrically adjusted to approximate the exact solution for a given Hamiltonian. The Rayleigh–Ritz theorem^78,79^ formulated in eq 10, ensures that for any arbitrary trial wave function, the expectation value of the Hermitian Hamiltonian with respect to the trial state is always greater than or equal to the ground state energy, *E*_0_, of that Hamiltonian, with closer states to the actual Hamiltonian ground state giving closer expectation values to the ground state energy. Therefore, in the VQE, the trial state should ideally be as physically accurate as possible to obtain accurate results. Mathematically, the Rayleigh–Ritz theorem for the variational method in quantum mechanics is formulated as follows:

10 $$E\left(\theta \right)=\frac{\left\langle \psi \left(\theta \right)\right|H\left|\psi \left(\theta \right)\right\rangle }{\left\langle \psi \left(\theta \right)\right|\psi \left(\theta \right)\rangle }\geq E_{0}$$

with **θ** being a vector of *n* real-valued parameters: θ_0_, θ_1_, ..., θ_*n*–1_. And since in our case, |ψ­(**θ**)⟩ is a normalized quantum state that satisfies

11 ⟨ψ(θ)|ψ(θ)⟩=1

we can simplify eq 10 as

12 $$E\left(\theta \right)=\left\langle \psi \left(\theta \right)\right|H\left|\psi \left(\theta \right)\right\rangle \geq E_{0}$$

Making use of eq 10, the VQE process begins by initializing a qubit register. Subsequently, a quantum circuit designed to simulate the physics and entanglements of |ψ­(**θ**)⟩ is applied to this register. We will refer to this quantum circuit as the ansatz. For the VQE to remain computationally feasible, the circuit depth of the ansatz–the maximum number of quantum gates applied sequentially–must be kept sufficiently low, therefore necessitating the use of a relatively compact ansatz. Once a good ansatz is chosen, the parameters **θ** are then classically varied iteratively until *E*(**θ**) is minimized. eq 10 ensures that the minimized energy will converge toward a value that is no lower than the Hamiltonian’s ground state energy.

The most expensive part of this procedure is the computation of *E*(**θ**) given a parameter vector **θ**, especially on a classical computer, as was discussed previously in the introduction. It is thus this computation that will be carried out on a quantum computer. A diagrammatic description of the full VQE procedure is given in Figure 2.

**Figure 2:**
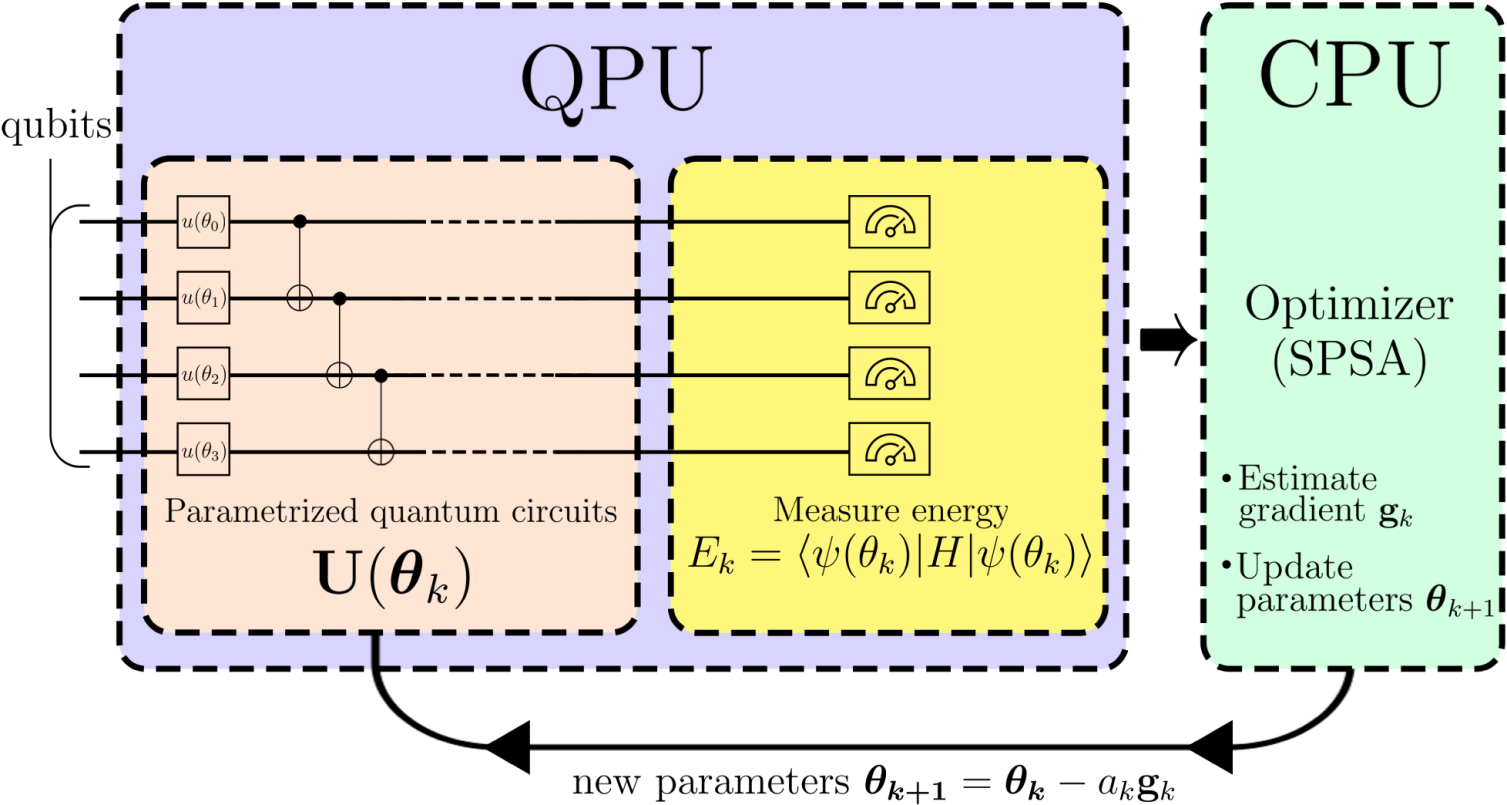
Iterative process and hybrid nature of the VQE. The quantum computer (QPU) is solely used for energy measurements, whereas the classical computer (CPU) is used for parameter optimization. We depict the SPSA as the optimization algorithm.

### 3.1 The Ansatz

When implementing the VQE for real quantum computers, we face the practical problem of choosing between accurate ansätze and noise-resilient ones. For quantum chemistry applications, this choice typically lies between the so-called *Hardware-Efficient Ansätze* (*HEA*s)^46,80^ that are primarily designed to be implementable on near-term quantum computers, or chemically inspired ansätze, such as the *Unitary Coupled-Cluster* (*UCC*) ansatz.^81,82^ *HEA*s aim to produce high-quality expectation values on noisy quantum computers but may not necessarily be physically informed. This renders the search space they have to cover larger than necessary. On the other hand, chemically inspired ansätze are designed to model electronic dynamics within the molecule and are thus more suitable for the variational principle under ideal conditions. Still, they are not guaranteed to achieve accurate results on current quantum computers due to their corresponding quantum circuits being deeper. Indeed, NISQ devices are constrained by factors such as noise, limited coherence times, gate fidelity, and qubit connectivity, all of which significantly limit their capability to execute complex or deep quantum circuits reliably. However, it is important to recognize that a shallower ansatz involving fewer quantum operations may lead to reduced accuracy in determining the ground state energy.

#### 3.1.1 Building a Hardware-Efficient Ansatz

*HEA*s form a broad class of ansätze, which are designed to be usable on near-term quantum computers.^40^ In this approach, unitaries are selected from a set of quantum gates guided by the connectivity and interactions inherent to the target quantum hardware. This method restrains the circuit’s depth increase typically associated with circuit *transpilation*, where we convert an arbitrary unitary into a sequence of gates that are native to the quantum computer. A key benefit of the hardware-efficient ansätze lies in their adaptability, as they allow for the encoding of symmetries^83^ and the closer alignment of correlated qubits for reduced depth, making it particularly advantageous for studying Hamiltonians that closely resemble the device’s native interactions.^84^ A widespread construction of *HEA*s is achieved by applying a layer of parametrized rotation gates on all the qubits, followed by a layer of entangling gates also acting on all the qubits.^46^ These rotations and entangling layer form a block that can be repeated *d* times. It is common for these circuits to begin and end with the rotation layer. Figure 3a,b shows two popular *HEA*s: the *Real Amplitudes*, and *Efficient SU2* ansätze, which have moderate expressive and entangling capabilities with a single layer.^46,80^ The former requires fewer rotations and parameters and produces quantum states with real coefficients, whereas the latter produces states with complex coefficients at the cost of additional gates and parameters.

**Figure 3:**
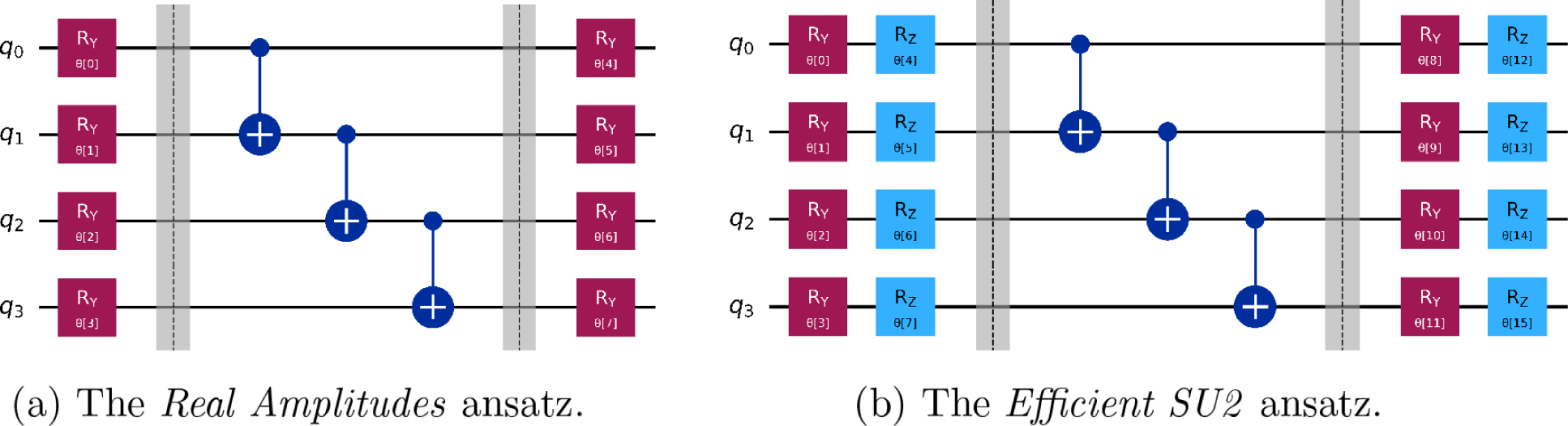
Quantum circuits for two *HEA*s: the *Real Amplitudes* and *Efficient SU2* ansätze. In this example, both ansätze act on four qubits. They are composed of a first rotation gates block, then an entangling block, then a final rotation gates block. The *Efficient SU2* circuit has double the amount of rotation gates since it produces states with complex-valued amplitudes.

An important factor to take into account when designing an *HEA* is the qubit connectivity of the target Quantum Processing Unit (QPU). This is due to the fact that entangling, or two-qubit, gates are less accurate than single-qubit gates, and entangling qubits that are not directly connected will require the use of expensive SWAP gates that will introduce additional noise during the computation.^85^ In this work, we adopt the *Efficient SU2* ansatz as our chosen *HEA*; thus, for the remainder of this manuscript, we will refer to it simply as the *Hardware-Efficient Ansatz*, or *HEA*. Figure 4 shows the difference between the initial logical *HEA* circuit shown in [Fig fig3]b and the final physical circuit. The latter is executed on the QPU, which in our case is IBM Fez.

**Figure 4:**
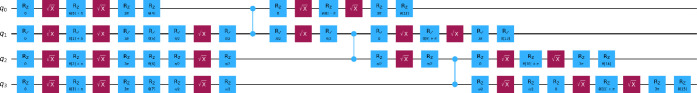
Transpiled *HEA* circuit that runs on the IBM Fez QPU. This circuit only uses gates that are physically implemented on the QPU, in this case, the $\sqrt{X}$ , R_Z_, and CZ gates. Therefore, the R_Y_ and CNOT gates have been decomposed. The qubits’ mapping has been kept the same since the initial CNOT gates already follow the target QPU’s connectivity.

#### 3.1.2 Building a Chemically-Inspired Ansatz

As stated above, an ideal ansatz for the implementation of the VQE in quantum chemistry would model molecular dynamics. A widely used model is the UCC theory, which describes the transitions of electrons from occupied orbitals to unoccupied ones while also modeling their correlations. In this section, we will use the following notation for the orbital indices: *i*, *j*, *k*, *l* for occupied orbitals; *m*, *n* for virtual (unoccupied) orbitals. This can be captured in the following ansatz,^81,82,86^

13 $$\left|\psi (\theta )\rangle =e^{\mathrm{T̂}(\theta )-{\mathrm{T̂}}^{†}(\theta )}|\psi_{\mathrm{init}}\right\rangle$$

where $\mathrm{T̂}\left(\theta \right)=\overset{n}{\underset{i=1}{\sum }}{\mathrm{T̂}}_{i}\;\left(\theta \right)$ is the cluster operator, which is a sum over *n* electron excitation operators ${\mathrm{T̂}}_{i}\;(\theta )$ . Each of these operators is written as

$${\mathrm{T̂}}_{i}\left(\theta \right)=\underset{k,m}{\sum }\theta_{k_{1}k_{2}\cdot \cdot \cdot k_{i}}^{m_{1}m_{2}\cdot \cdot \cdot m_{i}}a_{m_{i}}^{†}\cdot \cdot \cdot a_{m_{2}}^{†}a_{m_{1}}^{†}a_{k_{i}}\cdot \cdot \cdot a_{k_{2}}a_{k_{1}}$$ 14

For example, the one- and two-electron excitation operators are

15 $${\mathrm{T̂}}_{1}\left(\theta \right)=\underset{i,m}{\sum }\theta_{i}^{m}a_{m}^{†}a_{i}$$

16 $${\mathrm{T̂}}_{2}\left(\theta \right)=\underset{i,j,m,n}{\sum }\theta_{ij}^{mn}a_{m}^{†}a_{n}^{†}a_{i}a_{j}$$

**θ**, in this case, is thus the vector of parameters associated with all the possible electron transitions, which are themselves modeled by the creation and annihilation operators *a* ^†^ and *a*. Since this ansatz preserves the number of electrons, the initial state |ψ_init_⟩ is chosen to be one of the possible occupation states, preferably the Hartree–Fock reference state, |HF⟩. Because implementing the full *UCC* ansatz is not practical, at least not for near-term quantum computers, as it would require a very deep circuit that implements all excitation operators ${\mathrm{T̂}}_{i}$ , it is common only to consider the *single* and *double* excitation operators ${\mathrm{T̂}}_{1}$ and ${\mathrm{T̂}}_{2}$ . The resulting restricted ansatz is thus called the *Unitary Coupled Cluster Singles and Doubles* (*UCCSD*) ansatz, where *T̂*(**θ**) → *T̂*_*SD*_(**θ**) such as

17 $${\mathrm{T̂}}_{SD}\left(\theta \right)={\mathrm{T̂}}_{1}\left(\theta \right)+{\mathrm{T̂}}_{2}\left(\theta \right)$$

18 $$=\underset{i,m}{\sum }\theta_{i}^{m}a_{m}^{†}a_{i}+\underset{i,j,m,n}{\sum }\theta_{ij}^{mn}a_{m}^{†}a_{n}^{†}a_{i}a_{j}$$

To implement this *UCCSD* ansatz on a quantum computer, we must go through two essential steps: mapping and a Trotter-Suzuki decomposition, also known as Trotterization, the former having been discussed already in Section 2.2. Trotterization is the process of transforming an exponential of a sum of noncommuting operators {*O*_*i*_} into a product of exponentials of single operators:^87–89^

19 $$e^{O_{1}+O_{2}+\cdot \cdot \cdot }=\underset{n\to \infty }{\lim}{\left(e^{O_{1}/n}e^{O_{2}/n}\cdot \cdot \cdot \right)}^{n}$$

The quantum state evolution described in eq 13 indeed includes an exponential of a sum of noncommuting operators ${\mathrm{T̂}}_{i}$ and their adjoints. Explicitly:

20 $$\left|\psi \left(\theta \right)\rangle_{\mathrm{UCCSD}}=e^{{\mathrm{T̂}}_{1}(\theta )+{\mathrm{T̂}}_{2}(\theta )-{{\mathrm{T̂}}_{1}}^{†}(\theta )-{{\mathrm{T̂}}_{2}}^{†}(\theta )}\right|\psi_{\mathrm{init}}\rangle$$

The Trotterization of the evolution operator then gives

$$e^{{\mathrm{T̂}}_{1}(\theta )+{\mathrm{T̂}}_{2}(\theta )-{{\mathrm{T̂}}_{1}}^{†}(\theta )-{{\mathrm{T̂}}_{2}}^{†}(\theta )}=\underset{n\to \infty }{\lim}{\left(e^{\frac{{\mathrm{T̂}}_{1}(\theta )}{n}}e^{\frac{{\mathrm{T̂}}_{2}(\theta )}{n}}e^{\frac{-{{\mathrm{T̂}}_{1}}^{†}(\theta )}{n}}e^{\frac{-{{\mathrm{T̂}}_{2}}^{†}(\theta )}{n}}\right)}^{n}$$ 21

This Trotterization process can present some subtle challenges for near-term quantum computers for two reasons: the first is associated with the exponent *n* in eq 21, which should be very large in the exact *UCCSD* solution limit. This means that the circuit simulating the product of exponentials, in our case $e^{{\mathrm{T̂}}_{1}\left(\theta \right)/n}e^{{\mathrm{T̂}}_{2}\left(\theta \right)/n}e^{-{{\mathrm{T̂}}_{1}}^{†}\left(\theta \right)/n}e^{-{{\mathrm{T̂}}_{2}}^{†}\left(\theta \right)/n}$ , will be repeated *n* times, for which the execution time may exceed our qubits’ coherence time on the one hand, and which leads to an accumulation of noise effects and errors on the other. The second reason is the simulation of each exponential operator, which requires a number of entangling gates that is proportional to the Trotterization degree, *n*, the number of Pauli terms, and their average weight,^36,90,91^ as shown in Table 1. These two reasons render the implementation of the *UCCSD* ansatz quantum computationally expensive and susceptible to quantum noise and errors. However, it was also numerically shown that in simple molecular systems, a single Trotter step (degree *n* = 1) is sufficient for an accurate description of the ground state^46,92^ since the variational optimization can reduce the effect of the Trotterization error.^81^ We will thus restrict ourselves to a single Trotter step.

In Table 2, we highlight how the logical entangling gates (CNOT gates) are decomposed into a greater number of CZ gates in the transpiled physical circuit corresponding to the utilized quantum computer, further accumulating errors and noise.

**Table 2:** A Comparison of the Logical and Transpiled *UCCSD* Ansatz and the *HEA* Circuits to Be Run on Real Hardware in Terms of Depth, Number of Entangling Gates (CNOT and CZ Gates), and Number of Parameters^a^,^b^

Logical Circuits			
Ansatz	Depth	CNOTs	Parameters
UCCSD	315	172	8
HEA	7	3	16

Transpiled Circuits			
Ansatz	Depth	CZs	Parameters
UCCSD (unoptimized)	1258	256	8
UCCSD (optimized)	615	185	8
HEA (unoptimized)	27	3	16
HEA (optimized)	21	3	16

a We consider both unoptimized and optimized transpilations with Qiskit’s transpiler’s optimization levels 0 and 3, respectively.

b The target real hardware here is the 156-qubit IBM Fez.

### 3.2 Optimization

Varying a set of values in order to minimize a function is a well-known classical procedure termed optimization. It is central to a variety of applications in science, engineering, and machine learning. A plethora of methods and tools for optimization have been developed to be used for a wide range of problems. In this setting, in particular, we are concerned with finding those values of the circuit parameters **θ** of the ansatz that minimize a cost function. This cost function is the expectation value of the molecular Hamiltonian with respect to the ansatz, and minimizing it corresponds to solving for the Hamiltonian’s ground state energy. This optimization problem is to be solved using classically implemented algorithms and can be posed as

22 $$\underset{\theta }{\min}E\left(\theta \right)=\left\langle \psi \left(\theta \right)\right|H\left|\psi \left(\theta \right)\right\rangle$$

where |ψ­(**θ**)⟩ is the state prepared by the parametrized ansatz, **θ** is a real-valued parameters vector, and *H* is the Hamiltonian operator that is to be measured.

After selecting an ansatz, it is crucial to choose a suitable optimizer, as this decision greatly influences both the convergence speed of the VQE optimization process and the overall computational cost of the algorithm, as well as the VQE’s resilience to noise in NISQ-era quantum computers. Below is a short description of one such method called the *Simultaneous Perturbation Stochastic Approximation* optimization.

#### 3.2.1 Simultaneous Perturbation Stochastic Approximation

The Simultaneous Perturbation Stochastic Approximation (SPSA) is an optimization method that was developed for applications that require optimizing a fluctuating, nondeterministic cost function.^66^ Although initially developed for purely classical applications, it has since proven useful for quantum computing, where it became a popular optimization method due to its performance in powering variational quantum algorithms under noisy conditions.^47^ The SPSA optimizer requires two energy measurements (cost function calls, in general) *E*(**θ**_*k*_ + *c*_*k*_ **Δ**_*k*_) and *E*(**θ**_*k*_ – *c*_*k*_ **Δ**_*k*_)^66^ to compute a gradient approximation. The component-wise gradient estimation is thus given by

23 $$g_{k,i}\left(\theta_{k}\right)=\frac{E(\theta_{k}+c_{k}\Delta_{k})-E(\theta_{k}-c_{k}\Delta_{k})}{2c_{k}\Delta_{k,i}}$$

where **θ**_k_ is the vector representing the current set of parameters (at iteration *k*), **Δ**_*k*_ is a random vector used to “perturb” the current parameters **θ**_*k*_, and *c*_*k*_ is a decaying scalar sequence used to attenuate the perturbations as the number of iterations *k* grows. After the approximate gradient vector **g**_*k*_ is computed, the next set of parameters is then updated to

24 $$\theta_{k+1}=\theta_{k}-a_{k}g_{k}$$

where *a*_*k*_ is also a scalar sequence that decays with *k*, called the learning rate. The procedure of estimating **g**_*k*_ and calculating **θ**_k+1_ is repeated until the VQE converges towards a minimum of the cost function. Usually, SPSA starts every optimization with a calibration step, which determines the appropriate learning rate sequence *a*_*k*_ depending on how much the cost function fluctuates. This calibration step requires a number of random cost function evaluations, often set to 50. Finally, *a*_*k*_ and *c*_*k*_ are given by

25 $$a_{k}=\frac{a}{{\left(A+k\right)}^{\alpha }}$$

and

26 $$c_{k}=\frac{c}{k^{\gamma }}$$

where α, γ, *A*, and *c* are tunable hyperparameters.^66,77,93^

## 4 Simulations and Quantum Hardware Implementation

### 4.1 Simulations

In this section, we analyze the behavior and convergence of the VQE for BeH_2_ under ideal and noisy conditions by means of classical simulations of quantum circuits. We will use the two ansätze we introduced above: the *HEA* and the chemically motivated *UCCSD*. Classically simulating downscaled versions of the VQE is a good first step to take in order to perform benchmarking and analysis, as well as initial debugging, as classical computing resources are cheaper and easier to access than their quantum counterparts. The Hamiltonian we are using is that of the BeH_2_ at a Be–H bond distance of 1.326 Å, with a Complete Active Space (CAS) approximation that includes 2 electrons and 3 active molecular orbitals. In our case, and since we are already dealing with a small-scale VQE, we will be simulating the same quantum circuits to be run on the quantum hardware later.

In the following experiments, we perform 30 VQEs for each ansatz and simulator. The initial states associated with the *HEA* and *UCCSD* are |0000⟩ and |HF⟩, respectively. The initial parameter vectors, **θ**, are randomized for each VQE run. For the parameters optimization, we use SPSA with the following hyperparameters: α = 0.602, γ = 0.101, *A* = 0, and *c* = 0.2.^77,93^ The optimization procedure starts with an initial 50 cost function calls to calibrate SPSA’s learning rate series *a*_*k*_, while the perturbation series, *c*_*k*_, is determined from the aforementioned hyperparameters. We chose to cut off the optimizer, and therefore the VQE, after 400 iterations in the simulations. In total, we will thus perform 1251 measurements: 50 for the calibration phase, 2 × 400 (gradient estimation) + 400 (energy measurement) for the optimization, and 1 final energy measurement. Lastly, each energy measurement is obtained using 4096 shots, that is, by measuring every quantum circuit 4096 times and computing the energy from the distribution of measurement results. All simulations ran on Qiskit 1.2.0 and Qiskit IBM Runtime 0.28.0.^94^

#### 4.1.1 Ideal Device Simulator

An *ideal simulator* (or *ideal device*) is an idealized quantum computer that is not affected by any noise channel such as decoherence, gate errors, or readout errors and which is numerically simulated on a classical machine. It may, however, be subject to what is called *shot noise*: fluctuations in the measurements that are due to probabilistic sampling around the classically computed expectation values. This simulates the nondeterministic nature of quantum measurements, even in the idealized case. In this work, the *ideal* VQEs are simulated with shot noise; that is, their measurement results are sampled from a probability distribution. We refer to this idealized device as the State-Vector Simulator (SVS). Since we have considered an approximated BeH_2_ electronic Hamiltonian, it is worth validating our VQE for both ansätze on an ideal device and analyzing their convergences toward the known ground state energy of the Hamiltonian. The target energy, in this case, is the minimum eigenvalue of the approximated Hamiltonian and is given by

27 $$E^{\mathrm{target}}=E_{0}=-15.56089\mathrm{Ha}$$

We can compute this target value by taking the mapped Hamiltonian in its matrix form and simply diagonalizing it numerically, which gives us the same result as the Full Configuration Interaction (FCI) method. This approach is generally inefficient but is not an issue for our small 4-qubit Hamiltonian.

In this ideal case, the *UCCSD* ansatz performs better than the *HEA*. Moreover, as is evident in Figure 5, we notice a faster convergence for the *UCCSD* compared to the *HEA*, with a small number of VQEs converging toward a local minimum situated at around −15.25 Ha. This validates both the *UCCSD* and the *HEA* as potentially good candidates for our molecular problem.

**Figure 5:**
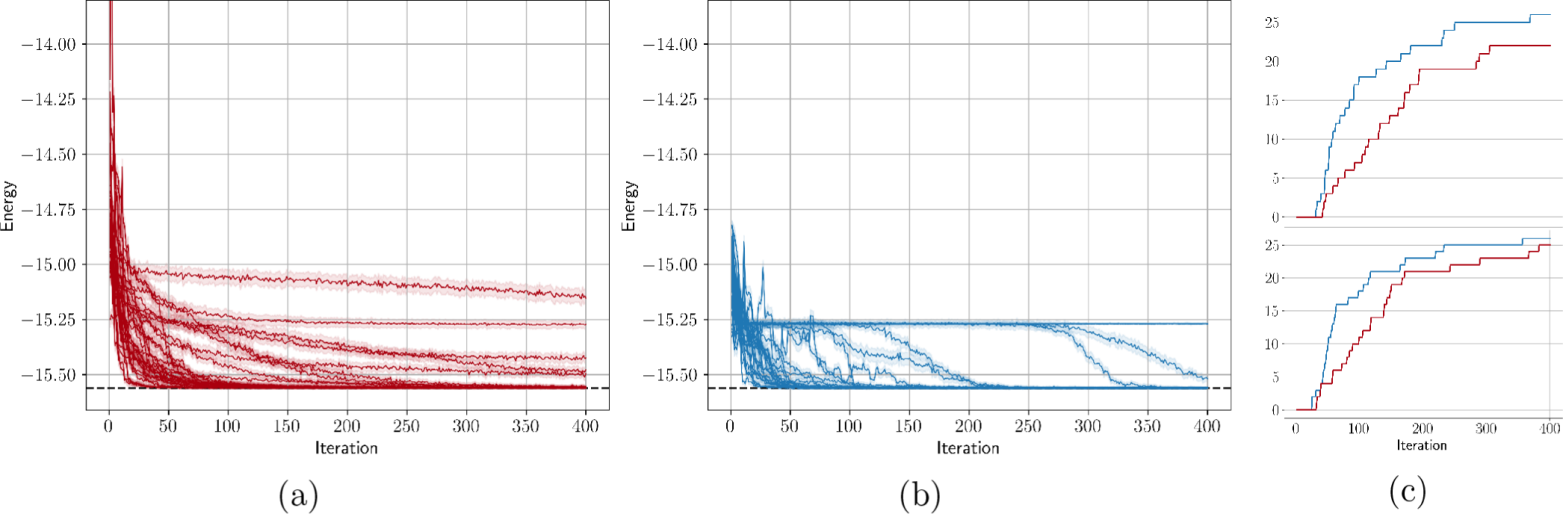
(a) *HEA* (red) and (b) *UCCSD* (blue) simulations on a perfect simulator with shot noise. The shaded area represents the standard deviation of each measurement, which results from 4096 measurement shots. Figures (c) top and bottom show the number of VQEs getting within 1 × and 3 × 1.6 mHa of *E* ^target^ at each iteration, respectively.

#### 4.1.2 Noisy Simulator

After validating that both ansätze converge within chemical accuracy (less than 1.6 mHa from *E* ^target^) in the ideal case, the next step is to investigate their performance when noise is introduced. This is done by simulating the noise profile of the target quantum device, as well as the device’s physical characteristics such as the qubits’ connectivity and the natively supported quantum gates. We chose to use noise models of the following IBM quantum computers: IBM Fez, IBM Torino, and IBM Strasbourg. The first two are of the Heron family, the latest IBM QPU family as of the time of writing, while the latter is of the precedent Eagle family. Heron QPUs have a higher number of qubits, lower noise levels, and a different 2-qubit entangling gate than Eagle QPUs.^95^ We specifically selected these three QPUs to showcase how the VQE results change mainly as a function of noise levels, with the least noisy QPU being IBM Fez, and the most noisy being IBM Strasbourg.

Table 2 compares the *UCCSD* ansatz and the *HEA* in terms of circuit depth and number of entangling gates as they would be implemented on the QPU, in addition to the number of parameters in each ansatz. In our simulations, we have used Qiskit’s optimization level 3 to transpile the *UCCSD* circuit. This is used for all the *UCCSD* VQEs.

Figure 6 shows the best noisy simulations graphs and Figure 7 shows the evaluation of energy values corresponding to the same best parameters on SVS, while in Table 3 we present the mean energy values over the last 10% iterations (40 in our case) for the best VQE results obtained on noisy simulators and the SVS energy evaluations of these best noisy results. We define the best result as the VQE with the lowest average energy over its last 10% of iterations. Interestingly, the two ansätze were affected differently by the introduced noise. VQEs with both the *UCCSD* and the *HEA* now converge toward a higher energy value compared to the ideal case, with the *HEA* performing significantly better. Furthermore, in Figures 6 and 7, as well as in the SVS results of Table 3, we observe that both ansätze ended up converging within less than 1 milliHartree (mHa) from the target energy *E* ^target^. However, the added noise affected the quality of the resulting optimized ansatz parameters much less than it affected the energy estimations, as is shown by the evaluation of these parameters on the SVS with no shot noise. Again, the *HEA* gave better results in this regard compared to *UCCSD*, although the *UCCSD* resulting optimized parameters are revealed to be much better than what the noisy energy estimates are indicating. These findings suggest that the VQE can be somewhat robust to the simulated levels of noise when it comes to parameter optimization, even if the measured energies are inaccurate.

**Figure 6:**
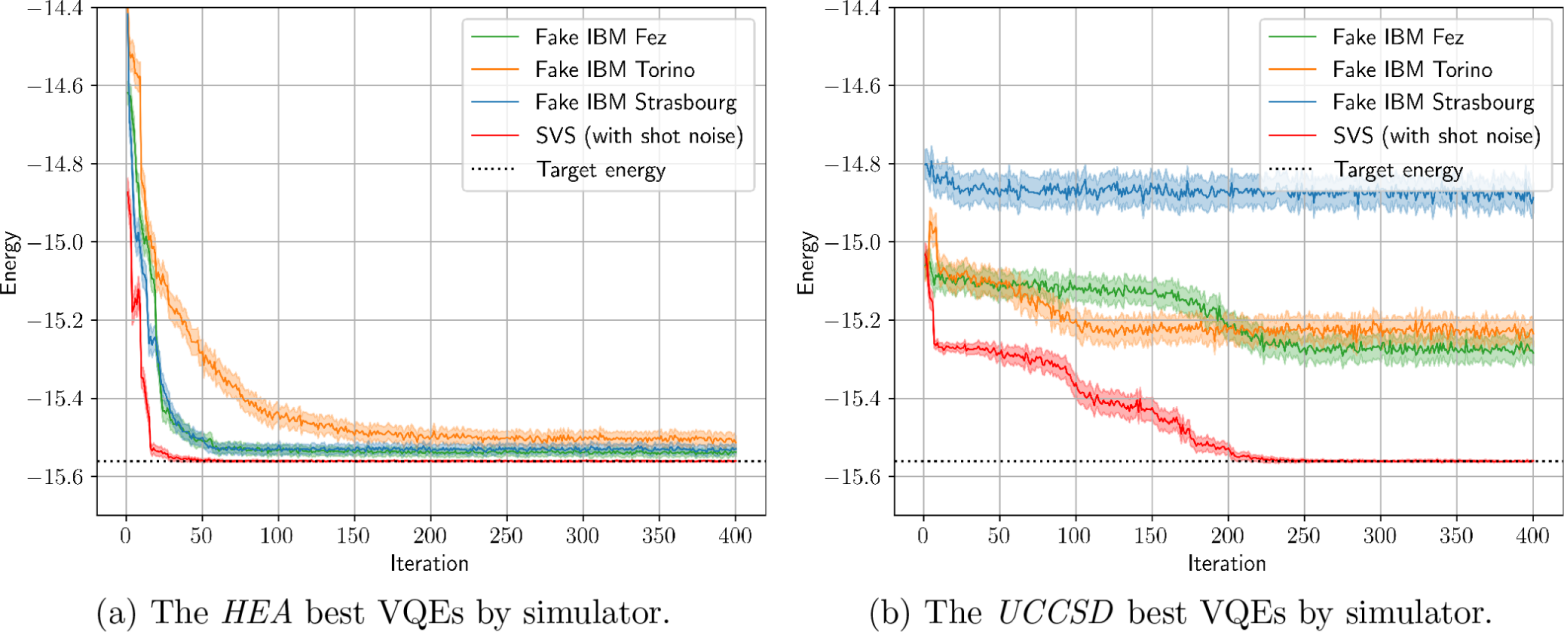
Convergence graphs of the best-performing VQE per simulator. For each simulator, the 30 VQEs are sorted by the average of their last 40 energies (last 10% of iterations). The VQEs with the lowest average are shown here. The shaded areas correspond to the standard deviation of each measurement (from 4096 shots).

**Figure 7:**
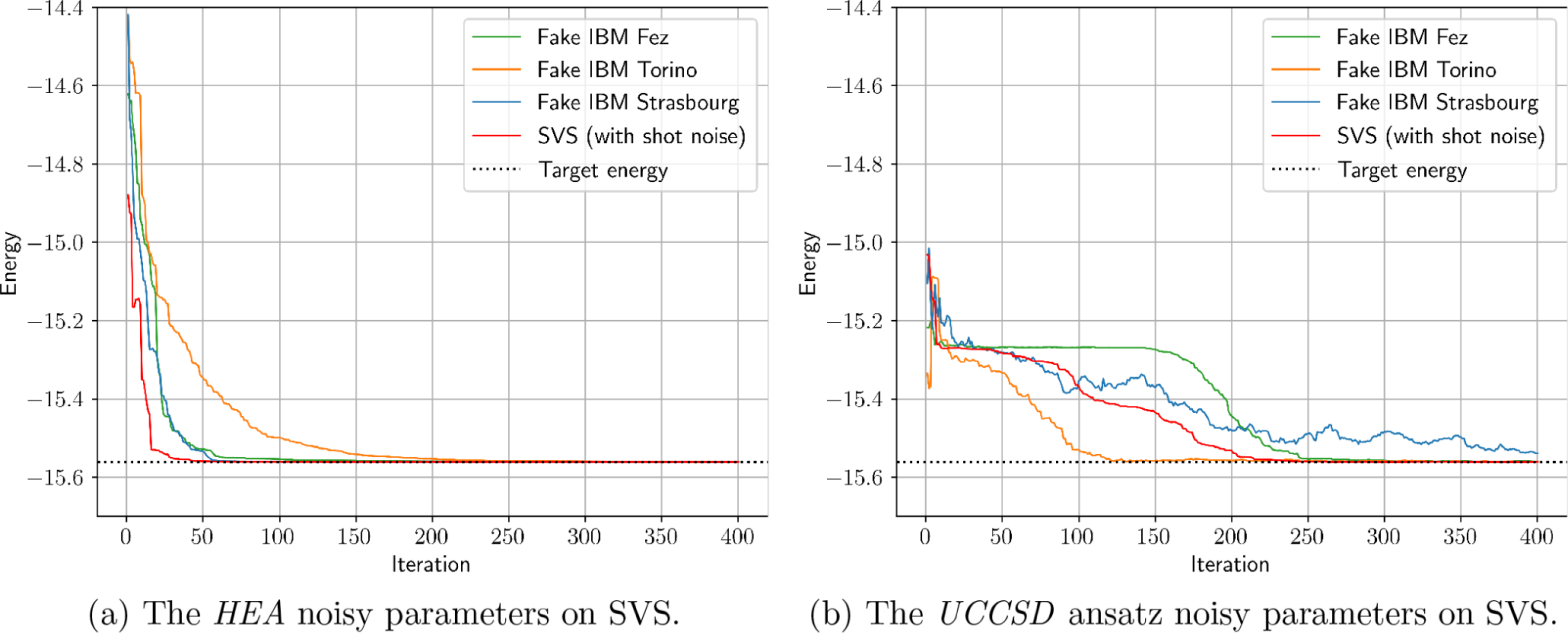
Convergence graphs with energies evaluated on SVS with no shot noise using the total set of parameters {**θ**_best_} of the best-performing VQE per ansatz and simulator.

**Table 3:** Mean Energy Value over the Last 10% Iterations (40), alongside the Standard Deviation Resulting from This Average for (a) Each Best VQE for Each Ansatz and Simulator, (b) the SVS-Evaluated Energies of Each Best VQE^a^,^b^,^c^

HEA			HEA on SVS		
Simulator	⟨*E*_VQE_({**θ**_best_}_last 10%_)⟩	Δ*E*_VQE_	Simulator	⟨*E*_SVS_({**θ**_best_}_last 10%_)⟩	Δ*E*_SVS_
IBM Torino	–15.50513(445)	0.05575	IBM Torino	**–15.55968(40)**	**0.00121**
IBM Strasbourg	–15.53111(291)	0.02978	IBM Fez	**–15.55993(14)**	**0.00096**
IBM Fez	–15.53934(243)	0.02155	IBM Strasbourg	**–15.56021(6)**	**0.00067**
SVS	**–15.56055(51)**	**0.00034**	SVS	**–15.56053(2)**	**0.00036**
*E* ^target^	–15.56089	-	*E* ^target^	–15.56089	-

UCCSD			UCCSD on SVS		
Simulator	⟨*E*_VQE_({**θ**_best_}_last 10%_)⟩	Δ*E*_VQE_	Simulator	⟨*E*_SVS_({**θ**_best_}_last 10%_)⟩	Δ*E*_SVS_
IBM Strasbourg	–14.87717(1304)	0.68371	IBM Strasbourg	–15.51147(1580)	0.04942
IBM Torino	–15.22978(1002)	0.33111	IBM Fez	–15.55865(136)	0.00224
IBM Fez	–15.27463(724)	0.28625	IBM Torino	–15.55916(77)	0.00173
SVS	**–15.56084(76)**	**0.00005**	SVS	**–15.56050(21)**	**0.00039**
*E* ^target^	–15.56089	-	*E* ^target^	–15.56089	-

a *E* ^target^ corresponds to the exact energy in the limit of the used level of theory, and Δ*E* = |⟨*E*⟩ – *E* ^target^|.

b Values within chemical accuracy (Δ*E* ≤ 0.0016 Ha) are in bold.

c All energies are in Ha.

### 4.2 QPU Experiment

We ran a VQE using the *HEA* on the IBM Fez QPU, described in Figure 8, for 180 iterations using the same setup that was described in previous subsections. The total computation time, including the SPSA calibration, classical pre-, postprocessing and optimization, communication, and quantum computations, was 5 h 30 m 39 s. The *quantum time*, defined as the amount of time a QPU spends on performing a quantum computation task,^95^ totaled 1 h 47 m 02 s. Table 4 summarizes the results of the VQE run on IBM Fez, Figure 9 shows the convergence graph of the experiment. The minimum energy that was measured on the QPU was $E_{\mathrm{QPU}}^{\min}=-15.45925(651)\mathrm{Ha}$ , at iteration 167, which when evaluated on SVS gives *E*_SVS_(**θ**_*k*=167_) = −15.55824 Ha. However, when we evaluate each iteration’s optimized parameters on SVS (without shot noise), we find that the best parameters are the ones produced at iteration 139, with an SVS-evaluated energy of *E*_SVS_(**θ**_*k*=139_) = −15.55901 Ha. For reference, these parameters gave on QPU an energy of *E*_QPU_(**θ**_*k*=139_) = −15.45790(650) Ha. The standard deviations given for the QPU energies are computed over the 4096 shots of the energy measurements. Finally, averaging over the last 10% of iterations (18 in this case) for the QPU-estimated and SVS-estimated energies gives ⟨*E*_QPU_(**θ**_QPU_)⟩ = −15.44416(879) Ha and ⟨*E*_SVS_(**θ**_QPU_)⟩ = −15.55824(26) Ha respectively, where the standard deviations result from averaging over the 18 last energy values.

**Figure 8:**
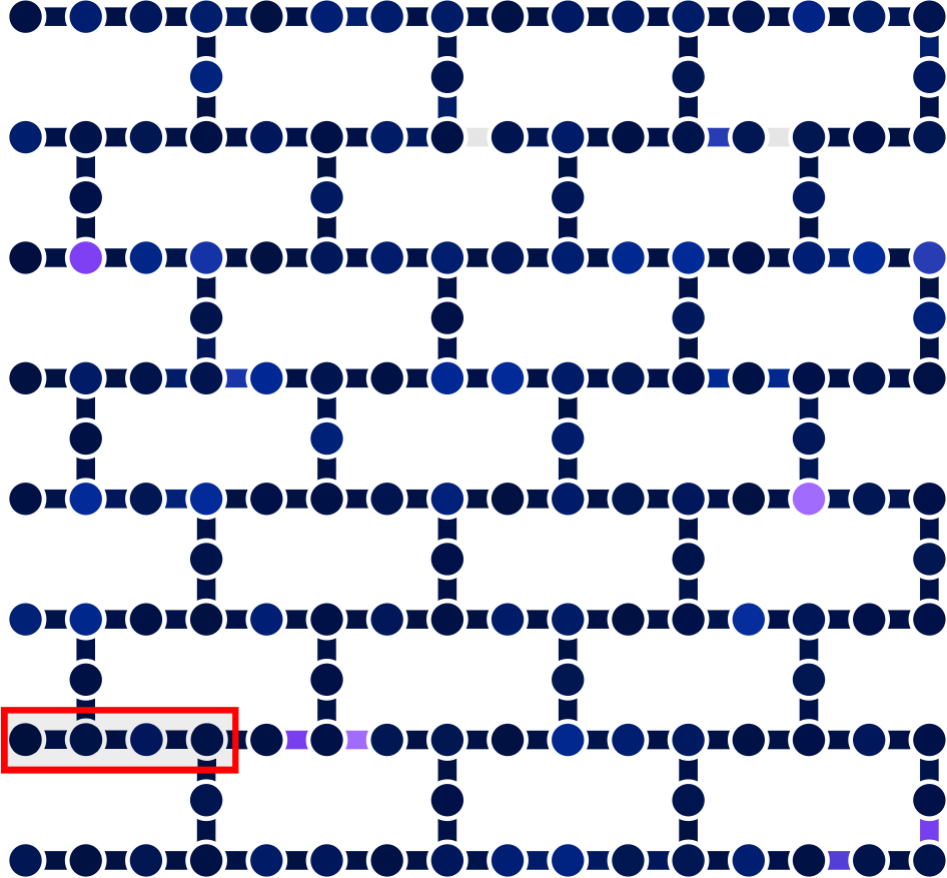
IBM Fez QPU’s qubit layout (vertices) and connectivity (edges). This QPU is of the Heron family, comprised of 156 qubits arranged in a heavy-hex lattice with cells of 12 qubits. The entangling gates are CZ gates. The used qubits, numbers 120 to 123, were manually selected based on their readout and CZ errors at the time of the VQE execution. The picture is adapted from IBM Quantum Web site^95^ in accordance with applying terms.

**Table 4:** Summary of the QPU experiment’s Results^a^,^b^,^c^,^d^

	*E*	Δ*E*	Iteration
min(*E*_QPU_)	–15.45925(651)	0.10164	167
min(*E*_SVS_)	–15.55901	0.00188	139
⟨*E*_QPU_⟩_last_ 10%	–15.44416(879)	0.11673	163–180
⟨*E*_SVS_⟩_last_ 10%	–15.55824(26)	0.00265	163–180

a We report the minimum energies on QPU and corresponding SVS evaluation as well as their iteration numbers.

b The standard deviations given in the upper and lower halves of the table result, respectively, from the energy measurements and the averaging over the last 10% of iterations.

c All energies are given in Ha, and Δ*E* = |*E* – *E* ^target^|.

d We also show the averages over the last 10% of iterations.

**Figure 9:**
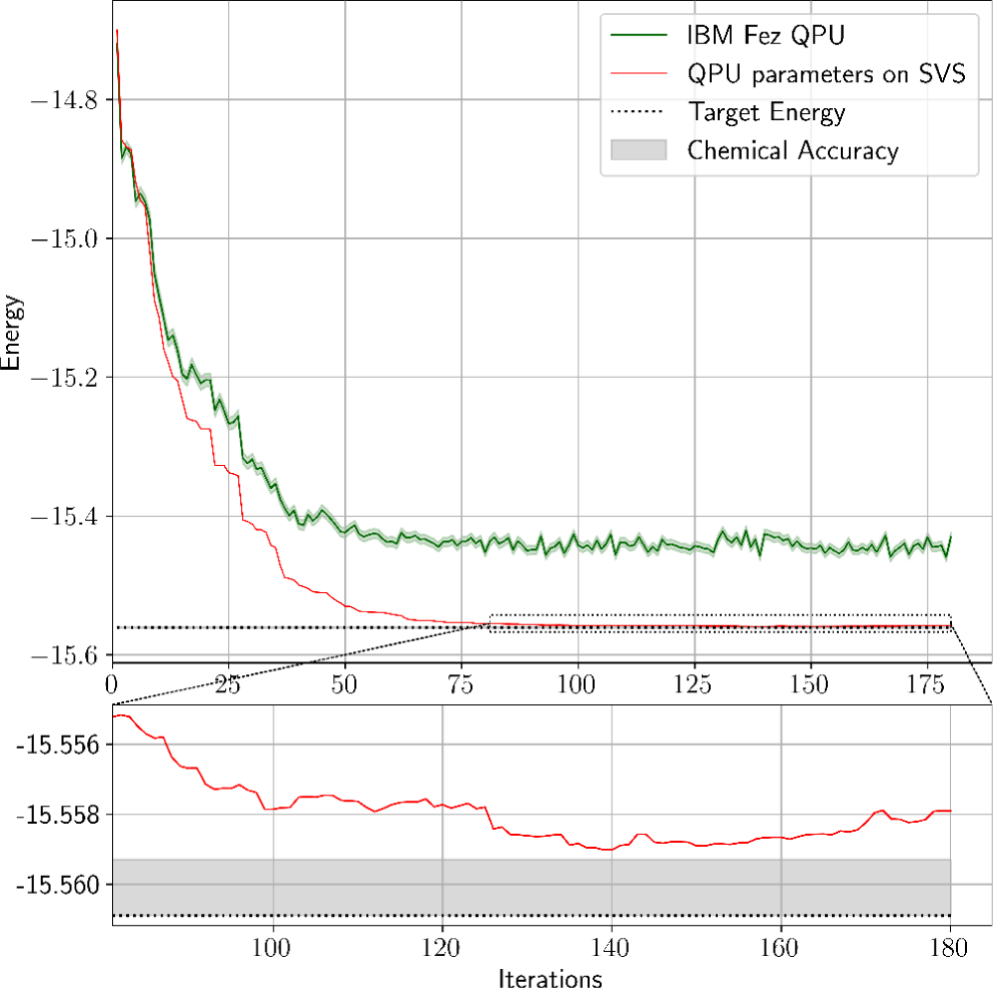
Results of the VQE run on IBM Fez (green). At each iteration, the optimized parameters are also evaluated on an SVS. The shaded area around the QPU energy graph is the standard deviation of the QPU measurements at 4096 shots. The corresponding SVS-evaluated energy graph (red) for the same optimized parameters is also shown.

#### 4.2.1 Error Mitigation

When running a VQE on actual QPUs or noisy simulators, the raw energy obtained may be far from the ideal result due to the cumulative effects of errors. However, Error Mitigation (EM) techniques, such as Zero-Noise Extrapolation (ZNE)^96,97^ readout/measurement error mitigation,^98^ Clifford data regression,^99^ Pauli Twirling,^100^ or probabilistic error cancellation, can significantly improve the accuracy of the results by reducing the impact of noise on the final outcome. To mitigate the effects of noise in the VQE’s results of this study, we applied the ZNE error mitigation technique after the VQE. In the ZNE technique, the noise in quantum computations is artificially amplified, and the results are extrapolated back to the zero-noise limit to estimate ideal noiseless outcomes. As error mitigation is not the focus of this work, the reader may refer to Giurgica-Tiron et al.^97^ for further details on the this method.

For our error mitigation step, we used the parameters corresponding to iteration 139–the iteration with the best SVS-evaluated energy, *E*_SVS_(**θ**_*k*=139_) = −15.55901 Ha. ZNE was carried out on the same QPU as the VQE, IBM Fez, using 40,000 shots per circuit, with integer noise-scaling factors (folds) 1, 3, and 5. The raw energy without any mitigation, *E*_raw_, or fold 1, was measured to be −15.49705 Ha, with an absolute error of Δ*E* = 61.96 mHa with respect to the above target energy. Table 5 summarizes the results of three extrapolations using a linear, quadratic, and exponential fitting functions. We find for our case that the quadratic extrapolation achieved the best accuracy with *E*_quad_ = −15.58634 Ha and Δ*E* = 27.33 mHa. Note that for the extrapolation procedures, we use the average measured energy values only without taking into account their standard deviations, and we thus report the ZNE results without standard deviations. Another point to take into consideration is that due to possible changes in the QPU’s noise characteristics between the VQE and ZNE experiments, the parameters, **θ**_*k*=139_, may yield different values in the VQE and ZNE measurements. Consequently, *E*_QPU_(**θ**_*k*=139_) with fold 1 was measured again at the same time as the other folds, so that all folds are affected by the same noise and device characteristics.

**Table 5:** ZNE-Mitigated Results Using the Parameters at 139^a^,^b^

Extrapolation	*E* (Ha)	Δ*E* (Ha)
*E*_raw_	–15.49705	0.06196
*E*_lin_	–15.69523	0.13622
*E*_quad_	–15.58634	0.02733
*E*_exp_	–15.60108	0.04207

a We show the results from three extrapolation methods: linear, quadratic, and exponential fittings.

b Δ*E* = |*E* – *E*_SVS_(**θ**_*k*__=139_)|, with *E*_SVS_(**θ**_*k*_=139) = −15.55901 Ha.

## 5 Discussion

In the context of the VQE, *ideal simulations* refer to the computation of the energy using a noiseless quantum circuit, typically carried out via a noiseless State-Vector Simulator (SVS). This method provides the theoretical ground state energy that would be obtained if all quantum gates and measurements were executed perfectly without any decoherence, gate errors, or readout errors. We do, however, simulate the fluctuations in quantum measurements, known as shot noise, in the SVS VQEs. We remind that the target energy for our molecular problem is *E* ^target^ = −15.56089 Ha, and we give here, for reference, the Hartree–Fock energy as *E* ^HF^ = −15.56033 Ha.

Noiseless simulations clearly demonstrate the reliability of the *Unitary Coupled-Cluster Single and Double* excitations (*UCCSD*) ansatz, with the majority of converging VQE instances reaching chemical accuracy (1.6 mHa from the target energy) after fewer iterations compared to the *Hardware-Efficient Ansatz* (*HEA*) as shown in Figure 5. Moreover, it is noteworthy that *UCCSD* provides an order of magnitude better average energy value (Δ*E* = 0.05 mHa) compared to the *HEA* (Δ*E* = 0.34 mHa), which highlights the efficiency of the chemically inspired UCC theory-based ansatz in the absence of noise. Additionally, in the absence of noise, both ansätze yield energy estimates within chemical accuracy of the target and below the Hartree–Fock (HF) energy, showcasing the effectiveness of the VQE under ideal, noiseless conditions.

However, real-world quantum computers introduce noise into the computation due to imperfections in gate operations, decoherence, environment-induced noise, and measurement errors. In noisy simulations, the energy measured is generally higher than the SVS energy, reflecting these additional imperfections. Therefore, comparing the ideal *E*_SVS_ with the energies obtained from noisy runs provides insight into optimization process under noise and the usefulness or limitations of current hardware. In this study we compared the computational accuracy at which the ground state energy of the BeH_2_ molecule can be estimated on three different quantum computer noise models for: IBM Strasbourg, Torino, and Fez. Each of these exhibiting distinct error rates. The effect of noise pushes the energy values above chemical accuracy by two and 4 orders of magnitude for the *HEA* and the *UCCSD*, respectively, when compared to the ideal device simulations. The difference becomes evident when comparing the performance of the *HEA* to the *UCCSD* ansatz. Errors are an order of magnitude higher for the *UCCSD* ansatz, independent of the noise model. This discrepancy is largely attributed to the significant difference in circuit depths between the two ansätze (see Table 2), highlighting the better noise-resilience of the *HEA* and emphasizes *UCCSD*’s sensitivity to hardware noise. Moreover, the absolute error across the three devices is of the order of 10^–2^ Ha for *HEA* but rises to the order of 10^–1^ Ha for *UCCSD*. This proves the greater robustness of *HEA* to hardware noise. It is also noteworthy that *UCCSD* exhibits larger measurement fluctuations in noisy simulations. Interestingly, the average energy evaluated on SVS over the set of the last 10% of parameters, {**θ**_best_}_last 10%_, for best performing noisy VQEs serves as a reference for what the variational ansatz could achieve under ideal conditions. When evaluated on SVS, all the results of the *HEA*-based VQE are within chemical accuracy from the energy target. Meanwhile, the *UCCSD* energy values remain beyond chemical accuracy. However, the error with respect to the target energy was reduced by 1 order of magnitude for IBM Strasbourg and 2 orders of magnitude for Torino and Fez. This shows the different effects of noise on the quality of the optimized parameters on one hand, and on the accuracy of the evaluated energy from the obtained VQE parameters on the other. A more in-depth analysis of VQE’s performance across different noise levels on the three noisy simulators we used is beyond the scope of this paper and will be addressed in future work.

In the light of the previous simulations, the results of the VQE implementation on IBM Fez, shown in Figure 9, are particularly interesting. The minimum energy that was measured on the QPU was $E_{\mathrm{QPU}}^{\min}=-15.45925\left(651\right)\mathrm{Ha}$ , corresponding to the parameters at iteration 167, **θ**_*k*=167_. When evaluated on SVS, these same parameters result in an evaluated energy *E*_SVS_(**θ**_*k*=167_) = −15.55847 Ha, higher than the target energy value by 2.24 mHa. However, evaluating the parameters **θ**_QPU_ for all iterations on SVS shows that a better parameter vector, **θ**_*k*=139_, has an energy *E*_SVS_(**θ**_*k*=139_) = −15.55901 Ha, a mere 1.88 mHa above the target energy. This finding indicates that we may optimize parameters well on QPU, despite misestimating their energies. Moreover, the average energy over the last 10% of iterations (18 in this case) for the SVS-evaluated energies is ⟨*E*_SVS_(**θ**_QPU_)⟩_last 10%_ = −15.55824(26) Ha, which is within the same range of 2× chemical accuracy from the target energy ([−15.56089, −15.55769] Ha). These SVS-evaluated energies draw a better picture of the quality of the solution produced by the VQE compared to the QPU-estimated energy value, and show that the VQE did converge to a good solution despite quantum noise and the larger error in the estimation of energy values by the QPU. The average QPU-estimated energy over the last 10% of iterations was ⟨*E*_QPU_(**θ**_QPU_)⟩_last 10%_ = −15.44416(879) Ha, again significantly higher than what the parameter vectors would give on SVS for the same iterations. This average also displays a larger standard deviation as noise amplifies the fluctuations in the energy estimation.

After applying error mitigation, we obtain a corrected energy value which serves as a more accurate approximation of the true ground state energy in the presence of noise. The mitigated energy should be regarded as one of the key results in assessing the success of the VQE experiment, as it reflects both the experimental realities of running quantum circuits on noisy hardware and the effectiveness of the error mitigation strategies employed.

In our work, we demonstrated the use of Zero-Noise Extrapolation (ZNE) on real quantum hardware to mitigate the measured QPU energy values. The error mitigation results presented in Table 5, and illustrated in Figure 10, show various degrees of improvements to the QPU-measured energy. The quadratic and exponential extrapolations improved upon the unmitigated QPU energy yielding respectively absolute errors Δ*E*_quad_ = 27.33 mHa, and Δ*E*_exp_ = 42.07 mHa. The linear extrapolation however produced a significantly worse error, Δ*E*_lin_ = 136.22 mHa. These results showcase the ability of methods such as ZNE to correct to a certain degree for the effect of noise on the quality of measured energies on noisy QPUs. This improvement is however not guaranteed. A poor choice of extrapolation methods, as was the case in the linear extrapolation for this specific case, will produce poor results. This, in particular, is one of the weaknesses of ZNE. Other techniques such as Clifford data regression aim to address these shortcomings, with challenges of their own.^99^

**Figure 10:**
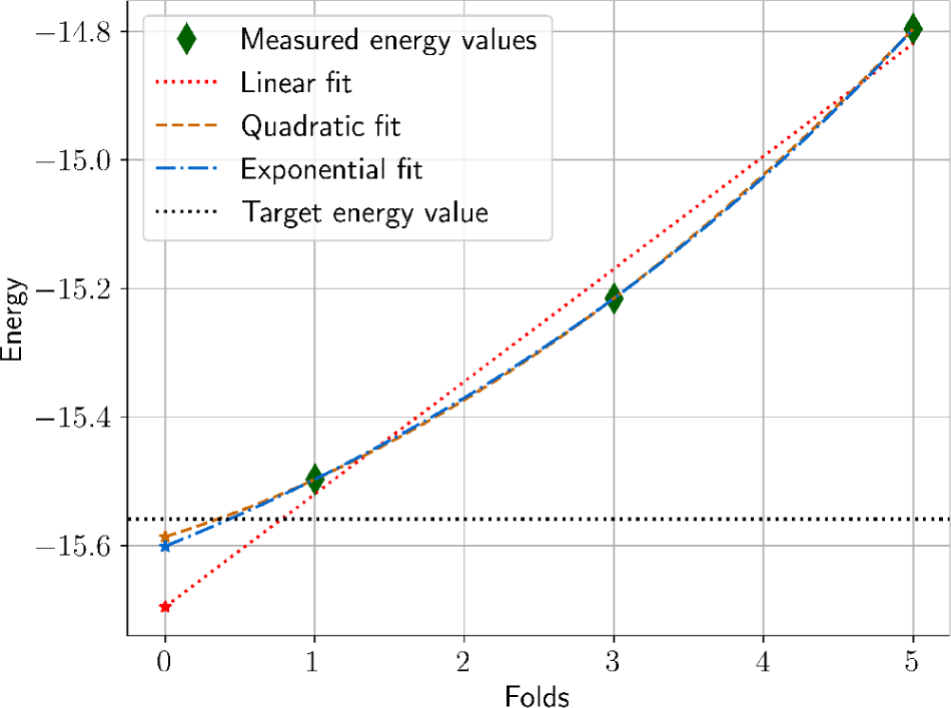
Zero-noise extrapolation results for the parameters of iteration 139. We show three fittings: linear, quadratic, and exponential, in addition to the zero-noise extrapolations (stars) at *x* = 0.

## 6 Conclusion

We have presented a self-contained study of the Variational Quantum Eigensolver (VQE) to estimate the ground state energy of the BeH_2_ molecule on a real Quantum Processing Unit (QPU). Comparing two ansätze from two different families across different noise conditions including three different noise models up to the real QPU.

Our algorithm, run on both ideal and noisy simulators, as well as on a real quantum device, successfully converges toward the target energy estimated from classical calculations within a reasonable number of iterations without requiring error mitigation during the VQE implementation. This work aims to provide a theoretical background and to provide essential tools for the simulation of larger molecules using the VQE. To demonstrate the effectiveness of the VQE on currently available quantum hardware, we performed energy calculations using noiseless simulators and noisy simulators based on the characteristics of three IBM quantum devices, each with distinct error rates: IBM Strasbourg, Torino, and Fez. Additionally, we carried out computation on the IBM Fez quantum computer, the most advanced device available to us with the lowest noise level, and consisting of 156 qubits.

Our study presents a comparative analysis of two conceptually different ansätze: the chemically inspired *UCCSD* and a hardware-efficient ansatz (*HEA*). While *UCCSD* achieves a higher accuracy on the state-vector simulator (SVS), it is significantly more sensitive to noise, making it less suitable for current NISQ devices. In contrast, the *HEA* exhibits promising performance across all platforms SVS, noisy simulators, and actual quantum hardware. Notably, *HEA* effectively optimizes parameters, achieving energy estimates within chemical accuracy relative to the exact solution at the level of the employed theory on SVS. Additionally, across all noisy simulations, *HEA* remains robust, and on quantum processing units, it produces optimized ground states corresponding to an exact energy estimate only 1.88 mHa above the target energy, even without the application of error mitigation (EM) techniques.

Indeed, while error mitigation techniques have proven highly effective in enhancing the accuracy of the VQE on current quantum computers, they often come at the cost of significantly increased resource demands. It remains uncertain whether this added resource requirement will be a manageable trade-off or a critical limitation as the VQE is scaled to larger, more complex applications. Our findings demonstrate that achieving ground state energy within chemical accuracy, compared to the exact solution at the chosen level of theory, is feasible without needing error mitigation during the VQE convergence. Applying EM as a postprocessing step can significantly reduce the computational resources required.

We have also elaborated on the mathematical framework for the Unitary Coupled Cluster with Single and Double excitations (*UCCSD*) and provided utilized an updated methodology for implementing the VQE using the latest version of Qiskit (1.2), as of the time of writing, employing the Simultaneous Perturbation Stochastic Approximation (SPSA) as classical optimizer. This approach aims to balance the theoretical rigor and depth found in review articles with practical applicability, ensuring a thorough yet accessible guide for researchers and practitioners.

Furthermore, state-vector energy estimations using the quantumly optimized parameters confirm that current quantum devices are effective in optimizing circuit parameters despite their tendency to misestimate the actual values of simulated energies. Similar results were reported by Sorourifar et al.^48,101^ using Bayesian optimization, while we observe this trend with SPSA in our study. The higher accuracy in estimating the energy landscape features over energies themselves thus appears independent of the optimizer used. This insight further advocates the VQE’s suitability for NISQ-era devices and its potential integration with novel algorithms such as the quantum sample-based diagonalization.^62–65^ We plan to explore this observation further in future works.

## Supplementary Material



## Notes

Additional quantum chemistry details; construction of molecular orbitals using LCAO and Gaussian primitives; building second-quantized Hamiltonians; computation of one- and two-electron integrals for H_2_; documented VQE implementation codes with Qiskit 1.2 (PDF)
