# Supplementary material for: Ground-State Energy Estimation on Current Quantum Hardware through the Variational Quantum Eigensolver: A Practical Study
Source: J Chem Theory Comput. 2025 Jun 29;21(14):6777–92. doi: 10.1021/acs.jctc.4c01657 (PMC12288014; doi:10.1021/acs.jctc.4c01657)
Supplement: Supplementary file 1 [file ct4c01657_si_001.pdf]

Supplementary Information for: Ground State Energy Estimation on Current Quantum Hardware Through the Variational Quantum Eigensolver: A Practical Study

Nacer Eddine Belaloui,[*,†,‡] Abdellah Tounsi,[†,‡] Abdelmouheymen Rabah Khamadja,[†,‡] Mohamed Messaoud Louamri,[†,¶] Achour Benslama,[†,‡] David E. Bernal Neira,[§] and Mohamed Taha Rouabah[*,†,‡]

†*Constantine Quantum Technologies, Frères Mentouri University Constantine 1, Ain El Bey Road, Constantine, 25017, Algeria*

‡*Laboratoire de Physique Mathématique et Subatomique, Frères Mentouri University Constantine 1, Ain El Bey Road, Constantine, 25017, Algeria*

¶*Theoretical Physics Laboratory, University of Science and Technology Houari Boumediene, BP 32 Bab Ezzouar, Algiers, 16111, Algeria*

§*Davidson School of Chemical Engineering, Purdue University, 480 Stadium Road, West Lafayette, IN, 47907, USA*

E-mail: nacer.belaloui@cqtech.org; m.taha.rouabah@umc.edu.dz

1 Molecular orbitals construction

To represent the spatial distribution of electrons in molecules, we need to choose an orthonormal basis set for the molecular orbital functions $\{\xi_p(\mathbf{r})|p = 1 \cdots M\}$. In computational chemistry, it is convenient to construct these molecular orbitals based on our knowledge of atomic orbitals $\{\phi_\alpha(\mathbf{r})|\alpha = 1 \cdots M\}$. In the Linear Combination of Atomic Orbitals (LCAO) method, molecular orbitals ξ_p are expressed as a linear combination of atomic orbitals ϕ_α. Each atomic orbital has a real coefficient $c_{p\alpha}$ that represents its contribution to the molecular orbital. Each molecular orbital is then expressed as

$$\xi_p(\mathbf{r}) = \sum_\alpha c_{p\alpha}\phi_\alpha(\mathbf{r}). \tag{1}$$

However, since it is hard to compute the electron integrals, especially the two-electron integrals, with Hydrogen-like atomic orbitals that have a Slater determinant form, it is more convenient to approximate these orbitals with a linear a combination of normalized primitive Gaussian functions[1] which take the form

$$\sigma_c(x, y, z) = N_c(x - R_x)^i(y - R_y)^j(z - R_z)^k \exp^{-\alpha_c(\mathbf{r}-\mathbf{R})^2}, \tag{2}$$

where i, j, k are non-negative integers and the orbital number $l = i + j + k$ specifies the shell type of the spherical part of the wave-function. The normalization factor N_c is given by[1,2]

$$N_c = \left(\frac{2\alpha_c}{\pi}\right)^{3/4} \left(\frac{(8\alpha_c)^{i+j+k} i!j!k!}{(2i)!(2j)!(2k)!}\right)^{1/2}. \tag{3}$$

The atomic orbitals are thus approximated as follows:

$$\phi_\alpha(\mathbf{r}) = \sum_c d_{\alpha c}\sigma_c(\mathbf{r}). \tag{4}$$

While the coefficients d_{ac} in Eq. (4) and the exponents α_c in Eq. (2) and Eq. (3) are determined to approximate the conventional atomic orbitals and preserve the normalization, the $c_{\mu a}$ coefficients in eq. (1) are computed with the Self-Consistent Field (SCF) method where the mean field energy of the molecule is minimized to get the Hartree-Fock state and the coefficients of the orbitals.[2,3] Basically, the orbital wave functions are written as a linear combination of the basis set as follows:

$$\xi_p(\mathbf{r}) = \sum_{\alpha c} c_{p\alpha} d_{\alpha c}\sigma_c(\mathbf{r}). \tag{5}$$

However, since electrons are spin 1/2 particles, their wave function should include a spin factor $\alpha(\mathbf{r})$ or $\beta(\mathbf{r})$ for a spin up or down, respectively. Therefore, the final form of the spin molecular orbitals is:

$$\psi_p(\mathbf{x}) = \psi_p(\mathbf{r}, s) = \begin{cases} \xi_p(\mathbf{r})\alpha(\mathbf{r})_{s=\uparrow} \\ \xi_p(\mathbf{r})\beta(\mathbf{r})_{s=\downarrow} \end{cases} \tag{6}$$

Taking into consideration the fermionic statistics of electrons, the Hartree-Fock method gives the wave function of the electronic ground state $\Psi(\mathbf{x_1}, \cdots, \mathbf{x_N})$ as the Slater determinant of the spin molecular orbitals $\{\psi_p(\mathbf{x}_i)|p = 1, \cdots, 2M; i = 1, \cdots, N\}$ for N electrons and M molecular orbitals such as $2M > N$.

Choosing a basis set is crucial to get an accurate estimation of the ground state energy.[4] The most accurate results can be achieved by considering all combinations of interactions between electrons in different molecular orbitals, all possible Slater determinants, and large basis set expansions.[3,5] However, this leads to more Hamiltonian terms and requires vast computational resources. There exists a diversity of basis sets in the quantum chemistry literature, each offering advantages and disadvantages that depend on the nature of the molecule to be studied. We cite three examples here which are suitable for small, medium, and large molecules :

- Minimal-Basis Sets: the STO-3G basis set[1,6,7] is one of the simplest options that is widely used for small molecules. It is a linear combination of three Gaussian functions of the form $d \cdot \exp(-\alpha \mathbf{r}^2)$ that produces Slater-type orbitals. The orbitals are distributed over the conventional shells. For the first row of atoms, we only have the $1s$ orbital. For the second row atoms, we have the $1s$ for the first shell and the $2s$ & $2p$ orbitals for the second shell. In general, the orbitals that share the same shell are given the same Gaussian exponents. For more accuracy, it is possible to use an STO-nG basis set, with a $n > 3$, which uses a linear combination of n Gaussian functions for each orbital. The minimal-basis sets give reliable results within short computational times, still, the accuracy not enough for molecules with more electrons and atoms.

- Small and Medium Basis Sets: the 3-21G, 3-21G$^{(*)}$, and 6-31G basis sets, or X-YZG,[1,7] are used for medium-sized molecules. These basis sets utilize two sets of functions, one for the core orbitals (X Gaussian functions), while each valence orbital is split into an inner one with Y Gaussian functions and an outer orbital with Z Gaussian functions (one in the above examples). Such medium basis sets provide more accuracy than STO-3G but will require more computational power. The previous two basis set types are introduced by Pople and his group.[1]

- Correlation-Consistent Basis Sets: the cc-sets are larger sets that are suitable for accurate chemical computations; they are called cc$pVXZ$ and first introduced by Dunning,[7,8] where p stands for polarization functions, V for valence, X for the number of shells the valence functions are split into, and Z for zeta. For instance, cc-pVTZ means correlation-consistent polarized valence triply-split zeta. These sets are more computationally demanding than either STO-nG and X-YZG but are significantly more accurate.

2 Building the Second Quantized Form

Since electrons are indistinguishable, we do not care which electron occupies which orbital. The second quantized states involve only information about the occupied orbitals. We start from the vacuum state $|\text{vac}\rangle$ where all orbitals are not occupied and there is no electron. Then, we start filling orbitals by creating electrons using the creation operators $a_p^\dagger$ for each spin molecular orbital ψ_p. Namely, $a_p^\dagger |\text{vac}\rangle = |\psi_p\rangle$. However, the creation of two electrons in the system implies an anti-symmetric state given by the Slater determinant:

$$a_q^\dagger a_p^\dagger |\text{vac}\rangle = \frac{1}{\sqrt{2}} \left(|\psi_p\rangle |\psi_q\rangle - |\psi_q\rangle |\psi_p\rangle \right). \tag{7}$$

The state $|\psi_q\rangle |\psi_p\rangle$ simply means that the first electron is in the state $|\psi_p\rangle$ while the second electron is in the state $|\psi_q\rangle$. Hence, the creation operators should obey the following algebra:

$$\{a_p^\dagger, a_q^\dagger\} = 0, \tag{8}$$

and the same for the annihilation operators a_p and a_q. Thus, the fermionic statistics are obeyed. The algebra of ladder operators is complete with :

$$\{a_p^\dagger, a_q\} = \delta_{pq}, \tag{9}$$

that accounts for Pauli's exclusion principle. Generally, we define the Fock state as

$$|n_0 n_1 \cdots n_k\rangle = (a_0^\dagger)^{n_0} (a_1^\dagger)^{n_1} \cdots (a_k^\dagger)^{n_k} |\text{vac}\rangle \,, \tag{10}$$

such that

$$n_i = \begin{cases} 1 & \text{if the } i\text{'th spin molecular orbital is occupied,} \\ 0 & \text{Otherwise} \end{cases} \tag{11}$$

The state $|n_0 n_1 \cdots n_k\rangle$ is a compact representation of a Slater determinant of all the occupied modes.

2.1 One-Electron Terms

The one-electron terms in the Hamiltonian are the kinetic term and the nucleus-electron Coulomb interaction term, that take the form:

$$\hat{F} = \sum_{i=1}^{N} \hat{f}(i). \tag{12}$$

such that $\hat{f}(i)$ is a function of the i'th electron's momentum and position operators. For each electron, the spin molecular orbitals form an orthonormal basis:

$$\textstyle\sum_p |\psi_p\rangle_i \, {}_i\langle\psi_p| = 1 \quad \forall \text{ electron } i. \tag{13}$$

Therefore, we can write the operator $\hat{f}(i)$ as

$$\hat{f}(i) = \sum_{pq} {}_i\langle\psi_p|\hat{f}(i)\,|\psi_q\rangle_i \, |\psi_p\rangle_i \, {}_i\langle\psi_q| \tag{14}$$

$$= \sum_{pq} f_{pq} \, |\psi_p\rangle_i \, {}_i\langle\psi_q|, \tag{15}$$

since ${}_i\langle\psi_p|\hat{f}(i)\,|\psi_q\rangle_i = f_{pq}$ what ever i is, the one-electron operators will be:

$$\hat{F} = \sum_{i=1}^{N}\sum_{pq} f_{pq}\,|\psi_p\rangle_{i\,i}\langle\psi_q| \tag{16}$$

$$= \sum_{pq} f_{pq}\sum_{i=1}^{N}|\psi_p\rangle_{i\,i}\langle\psi_q|. \tag{17}$$

It is possible to prove by involving an accurate correspondence between Fock and orbital states[9] that:

$$\sum_{i=1}^{N}|\psi_p\rangle_{i\,i}\langle\psi_q| = a_p^\dagger a_q. \tag{18}$$

Therefore,

$$\hat{F} = \sum_{pq} f_{pq} a_p^\dagger a_q. \tag{19}$$

And since the operator $\hat{f}$ terms are functions of momentum and position operators,

$$f_{pq} = \langle\psi_p|\,\hat{f}\,|\psi_q\rangle \tag{20}$$

$$= \int \psi_p^*(\mathbf{r})\hat{f}\psi_q(\mathbf{r})d\mathbf{r}. \tag{21}$$

2.2 Two-Electron Terms

The general form of a two-body operator, such as the Coulomb interaction between two electrons, can be written as

$$\hat{G} = \sum_{\substack{i,j=1\\ i>j}}^{N}\hat{g}(i,j). \tag{22}$$

Knowing that any two-body operator can be written as an expansion of the product of two one-body operators:

$$\hat{G} = \sum_{\substack{i,j=1\\ i>j}}^{N}\sum_{k} c_k \hat{f}^{\alpha_k}(i)\hat{h}^{\beta_k}(j) \tag{23}$$

$$= \sum_{k} c_k \sum_{\substack{i,j=1\\ i>j}}^{N}\hat{f}^{\alpha_k}(i)\hat{h}^{\beta_k}(j). \tag{24}$$

Using the results of one-electron operator and from the algebra of ladder operators:

$$a_p^\dagger a_s a_q^\dagger a_r = a_p^\dagger a_q^\dagger a_r a_s + \delta_{qr} a_p^\dagger a_s, \tag{25}$$

it is possible to show that

$$\hat{G} = \sum_{pqrs} g_{pqsr} a_p^\dagger a_q^\dagger a_s a_r \tag{26}$$

where

$$g_{pqsr} = {}_i\langle\psi_p|\, {}_j\langle\psi_q|\hat{g}(i,j)\,|\psi_s\rangle_j\,|\psi_r\rangle_i \quad \forall i,j. \tag{27}$$

Finally, we re-write the above as

$$g_{pqrs} = \int\int \psi_p^*(\mathbf{r_1})\psi_q^*(\mathbf{r_2})\hat{g}(1,2)\psi_s(\mathbf{r_2})\psi_r(\mathbf{r_1})d\mathbf{r_1}d\mathbf{r_2}. \tag{28}$$

3 Computing the one- and two-electron integrals for the H_2 molecule

In this appendix, we will compute, as an example, the one- and two-electron integrals for the H2 molecule with an interatomic distance of 0.74Å using the STO-3G basis set.

3.1 Molecular geometry of the H_2 molecule

The molecular geometry of the H_2 molecule is rather simple, as illustrated in Figure S1, it is

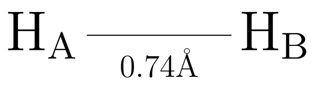

Figure S1: The molecular geometry of H_2.

only two hydrogen atoms separated by a 0.74Å bond distance at the equilibrium ground state; the first quantized electronic Hamiltonian for this molecule is given in the following form:

$$H_{el} = -\frac{\nabla_1^2}{2} - \frac{\nabla_2^2}{2} - \frac{1}{|\mathbf{R}_a - \mathbf{r}_1|} - \frac{1}{|\mathbf{R}_b - \mathbf{r}_1|} - \frac{1}{|\mathbf{R}_a - \mathbf{r}_2|} - \frac{1}{|\mathbf{R}_b - \mathbf{r}_2|} + \frac{1}{|\mathbf{r}_1 - \mathbf{r}_2|}. \tag{29}$$

It is worth noting that the Hamiltonian is in the atomic units. The one-electron integrals h_{pq} are defined as in the main paper's Eq. (3). Practically, since the spin states are orthonormal, we write these integrals in the molecular orbitals basis $\{\xi_p(\boldsymbol{r})\}$ in two parts as $h_{pq} = T_{pq} + V_{pq}$ such that:

$$T_{pq} = -\frac{1}{2}\int \xi_p(\mathbf{r})\nabla^2\xi_q(\mathbf{r})\,d\mathbf{r}, \tag{30}$$

$$V_{pq} = \sum_{c\in\text{nuclei}} \int \xi_p(\mathbf{r})\frac{-1}{|\mathbf{r} - \mathbf{R}_c|}\xi_q(\mathbf{r})\,d\mathbf{r}, \tag{31}$$

where T_{pq} and V_{pq} represent the contribution of the p and q molecular orbitals to the kinetic energy and the nuclear attraction energy, respectively. The two-electron integrals in the main paper's Eq.

(4) can be written in the molecular basis since the two spin states are orthonormal to each other as:

$$h_{pqrs} = \int\int \xi_p(\mathbf{r}_1)\xi_q(\mathbf{r}_2)\frac{1}{|\mathbf{r}_1 - \mathbf{r}_2|}\xi_r(\mathbf{r}_1)\xi_s(\mathbf{r}_2)d\mathbf{r}_1 d\mathbf{r}_2, \tag{32}$$

where $\xi_p(r)$ are the molecular orbitals' functions described as a LCAO as shown in Eq. (1). Working in the STO-3G basis, we define our atomic orbitals (AO) as a linear combination of three normalized Gaussian functions:

$$\phi_\alpha(\mathbf{r}) = \sum_{c=1}^{3} d_{\alpha c}\sigma_c(\mathbf{r} - \mathbf{R}_\alpha), \tag{33}$$

where the normalized Gaussian functions (2) are given in the case of s orbitals ($l = 0$) as:

$$\sigma_c(\mathbf{r} - \mathbf{R}_\alpha) = \left(\frac{2\alpha_c}{\pi}\right)^{3/4} \exp\bigl(-\alpha_c(|\mathbf{r} - \mathbf{R}_\alpha)|^2\bigr). \tag{34}$$

3.2 Kinetic Energy Integral Computation Over Atomic Orbitals in the STO-3G basis

The computation of kinetic energy integrals over primitive atomic orbitals in the STO-3G basis is performed using the following integral:

$$T_{pq} = -\frac{1}{2}\sum_{\alpha\beta=1}^{2} c_{p\alpha}c_{q\beta}\int \phi_\alpha(\mathbf{r})\nabla^2\phi_\beta(\mathbf{r})\, d\mathbf{r}, \tag{35}$$

where ϕ_a and ϕ_b are AO functions defined in Eqs. (1) and (33). In the STO-3G basis, the kinetic energy integral can be rewritten by developing the AO as a linear combination of Gaussians:

$$T_{pq} = \sum_{\alpha\beta=1}^{2}\sum_{ab=1}^{3} c_{p\alpha}c_{q\beta}d_{\alpha a}d_{\beta b}\langle a| - \frac{1}{2}\nabla^2|b\rangle, \tag{36}$$

where:

$$\langle a| - \frac{1}{2}\nabla^2|b\rangle = \int -\frac{1}{2}\sigma_a(\mathbf{r} - \mathbf{R}_a)\nabla^2\sigma_b(\mathbf{r} - \mathbf{R}_b)\, d\mathbf{r}. \tag{37}$$

The following computations of one- two-electron integrals over primitive Gaussian functions follow the methodology outlined in Szabo and Ostlund [5].

Evaluating $\langle a| -\frac{1}{2}\nabla^2|b\rangle$ is straight forward after letting ∇^2 operate, we utilize the Gaussian product theorem to express the two Gaussians as one centered in $\mathbf{R}_p$:

$$\sigma_a(\mathbf{r} - \mathbf{R}_a)\sigma_b(\mathbf{r} - \mathbf{R}_b) = N_p \exp\bigl(-\alpha_p|\mathbf{r} - \mathbf{R}_p|^2\bigr) \exp\left(-\frac{\alpha_a\alpha_b}{\alpha_a + \alpha_b}|\mathbf{R}_a - \mathbf{R}_b|^2\right), \tag{38}$$

where the new exponent $\alpha_p = \alpha_a + \alpha_b$ and $N_p = N_a N_b$ and $\boldsymbol{R_p}$ takes the form:

$$\mathbf{R}_p = \frac{\alpha_a\mathbf{R}_a + \alpha_b\mathbf{R}_b}{\alpha_a + \alpha_b}. \tag{39}$$

Simplifying and evaluating the integrals, $\langle a| - \frac{1}{2}\nabla^2|b\rangle$ takes the form:

$$\langle a| - \frac{1}{2}\nabla^2|b\rangle = N_a N_b \frac{\alpha_a \alpha_b}{\alpha_a + \alpha_b}\left[3 - \frac{2\alpha_a\alpha_b}{\alpha_a+\alpha_b}|\mathbf{R}_a - \mathbf{R}_b|^2\right]\left[\frac{\pi}{\alpha_a+\alpha_b}\right]^{\frac{3}{2}} \exp\left(-\frac{\alpha_a\alpha_b}{\alpha_a+\alpha_b}|\mathbf{R}_a - \mathbf{R}_b|^2\right). \tag{40}$$

We can get T_{pq} by summing over all contractions as shown in Eq. (36).

3.3 Nuclear Attraction Integral Computation Over Atomic Orbitals in the STO-3G basis

The computation of the nuclear attraction integrals over primitive Gaussian functions can be derived similarly to the kinetic energy integral. The integral to be evaluated is:

$$V_{pq} = \sum_{c\in\text{nuclei}} \sum_{\alpha\beta=1}^{2} c_{p\alpha} c_{q\beta} \int \phi_\alpha(\mathbf{r}) \frac{-1}{|\mathbf{r} - \mathbf{R}_c|} \phi_\beta(\mathbf{r})\, d\mathbf{r}. \tag{41}$$

We can develop the AO in the STO-3G basis to get:

$$V_{pq} = \sum_{c\in\text{nuclei}} \sum_{\alpha\beta=1}^{2} c_{p\alpha} c_{q\beta} \sum_{ab=1}^{3} d_{pa} d_{qb} \langle a| \frac{-1}{|\mathbf{r} - \mathbf{R}_c|} |b\rangle, \tag{42}$$

where

$$\langle a| \frac{-1}{|\mathbf{r} - \mathbf{R}_c|} |b\rangle = \int \sigma_a(\mathbf{r} - \mathbf{R}_a) \frac{-1}{|\mathbf{r} - \mathbf{R}_c|} \sigma_b(\mathbf{r} - \mathbf{R}_b) d\mathbf{r}. \tag{43}$$

Using the Gaussian product theorem, we define the new Gaussian centered in $\mathbf{R}_p$; the integral can then be written as:

$$\langle a| \frac{-1}{|\mathbf{r} - \mathbf{R}_c|} |b\rangle = -N_a N_b \exp\left(-\frac{\alpha_a\alpha_b}{\alpha_a+\alpha_b}|\mathbf{R}_a - \mathbf{R}_b|^2\right) \int \frac{\exp(-\alpha_p(|\mathbf{r} - \mathbf{R}_p)|^2)}{|\mathbf{r} - \mathbf{R}_c|} d\mathbf{r}. \tag{44}$$

It has been shown that

$$\int \frac{\exp(-\alpha_p(|\mathbf{r} - \mathbf{R}_p)|^2)}{|\mathbf{r} - \mathbf{R}_c|} d\mathbf{r} = \frac{2\pi}{\alpha_a + \alpha_b} F_0\left((\alpha_a+\alpha_b)|\mathbf{R}_p - \mathbf{R}_c|^2\right), \tag{45}$$

with $F_0(t)$ being the zeroth order Boys function, which relates to the error function as:

$$F_0(t) = \frac{1}{2}\sqrt{\frac{\pi}{t}}\text{erf}(\sqrt{t}), \tag{46}$$

with the interesting property of $\lim_{t\to 0} F_0(t) = 1$. The integral is then given by:

$$\langle a| \frac{-1}{|\mathbf{r} - \mathbf{R}_c|} |b\rangle = N_a N_b \frac{-2\pi}{\alpha_a + \alpha_b} \exp\left(-\frac{\alpha_a\alpha_b}{\alpha_a+\alpha_b}|\mathbf{R}_a - \mathbf{R}_b|^2\right) F_0\left((\alpha_a+\alpha_b)|\mathbf{R}_p - \mathbf{R}_c|^2\right). \tag{47}$$

We can get V_{pq} by summing over all contractions as shown in Eq. (42).

3.4 Two-Electron Integral Computation Over Atomic Orbitals in the STO-3G basis

The two-electron integral, as shown in Eq. (32), can be rewritten in AO basis as

$$h_{pqrs} = \sum_{\alpha\beta\gamma\delta=1}^{2} c_{p\alpha}c_{q\beta}c_{r\gamma}c_{s\delta} \int\int \phi_\alpha(\mathbf{r}_1)\phi_\beta(\mathbf{r}_1)\frac{1}{|\mathbf{r}_1 - \mathbf{r}_2|}\phi_\gamma(\mathbf{r}_2)\phi_\delta(\mathbf{r}_2)d\mathbf{r}_1 d\mathbf{r}_2, \tag{48}$$

which, after developing the AO on the STO-3G basis, is written as:

$$h_{pqrs} = \sum_{\alpha\beta\gamma\delta=1}^{2} c_{p\alpha}c_{q\beta}c_{r\gamma}c_{s\delta} \sum_{abcd=1}^{3} d_{\alpha a}d_{\beta b}d_{\gamma c}d_{\delta d}\langle ab|cd\rangle, \tag{49}$$

where $\langle ab|cd\rangle$ is given by

$$\langle ab|cd\rangle = \int\int \sigma_a(\mathbf{r}_1 - \mathbf{R}_a)\sigma_b(\mathbf{r}_1 - \mathbf{R}_b)\frac{1}{|\mathbf{r}_1 - \mathbf{r}_2|}\sigma_c(\mathbf{r}_2 - \mathbf{R}_c)\sigma_d(\mathbf{r}_2 - \mathbf{R}_d)d\mathbf{r}_1 d\mathbf{r}_2, \tag{50}$$

where $\sigma_a, \sigma_b, \sigma_c, \sigma_d$ are normalized primitive Gaussian functions defined above.
We use the Gaussian product theorem to reduce the two Gaussians on the left and the two Gaussians on the right; the new centers $\boldsymbol{R}_u$ and $\boldsymbol{R}_v$ are:

$$\mathbf{R}_u = \frac{\alpha_a\mathbf{R}_a + \alpha_b\mathbf{R}_b}{\alpha_a + \alpha_b}, \tag{51}$$

$$\mathbf{R}_v = \frac{\alpha_c\mathbf{R}_c + \alpha_d\mathbf{R}_d}{\alpha_c + \alpha_d}. \tag{52}$$

After further mathematical development, and using the Boys function, the integral takes this final form:

$$\langle ab|cd\rangle = N_aN_bN_cN_d\frac{(2\pi^2)^{\frac{5}{2}}\exp\left(-\frac{\alpha_a\alpha_b|\mathbf{R}_a-\mathbf{R}_b|^2}{\alpha_a+\alpha_b} - \frac{\alpha_c\alpha_d|\mathbf{R}_c-\mathbf{R}_d|^2}{\alpha_c+\alpha_d}\right)}{(\alpha_a+\alpha_b)(\alpha_c+\alpha_d)\sqrt{\alpha_a+\alpha_b+\alpha_c+\alpha_d}}F_0\left(\frac{(\alpha_a+\alpha_b)(\alpha_c+\alpha_d)}{\alpha_a+\alpha_b+\alpha_c+\alpha_d}|\mathbf{R}_u - \mathbf{R}_v|^2\right). \tag{53}$$

We can get h_{pqrs} by summing over all contractions as shown in Eq. (49).

4 Basic VQE pipeline in Qiskit 1.2

The VQE, as outlined in Ritz' variational principle, can be broken down into several critical components, each of which requires careful decisions that influence the algorithm's structure and computational cost. This sequence of components is often referred to as the VQE pipeline. Decisions made regarding individual elements within this pipeline can have critical effects on the entire VQE procedure. In Figure S2, we illustrate the iterative process, including the primary VQE loop, to provide a visual representation of the algorithm and its key components.

4.1 Defining the Molecular Problem

The `Qiskit` SDK[10] and its ecosystem of companion packages greatly simplify the task of implementing a full VQE pipeline, starting from a geometric description of a molecule to ending with

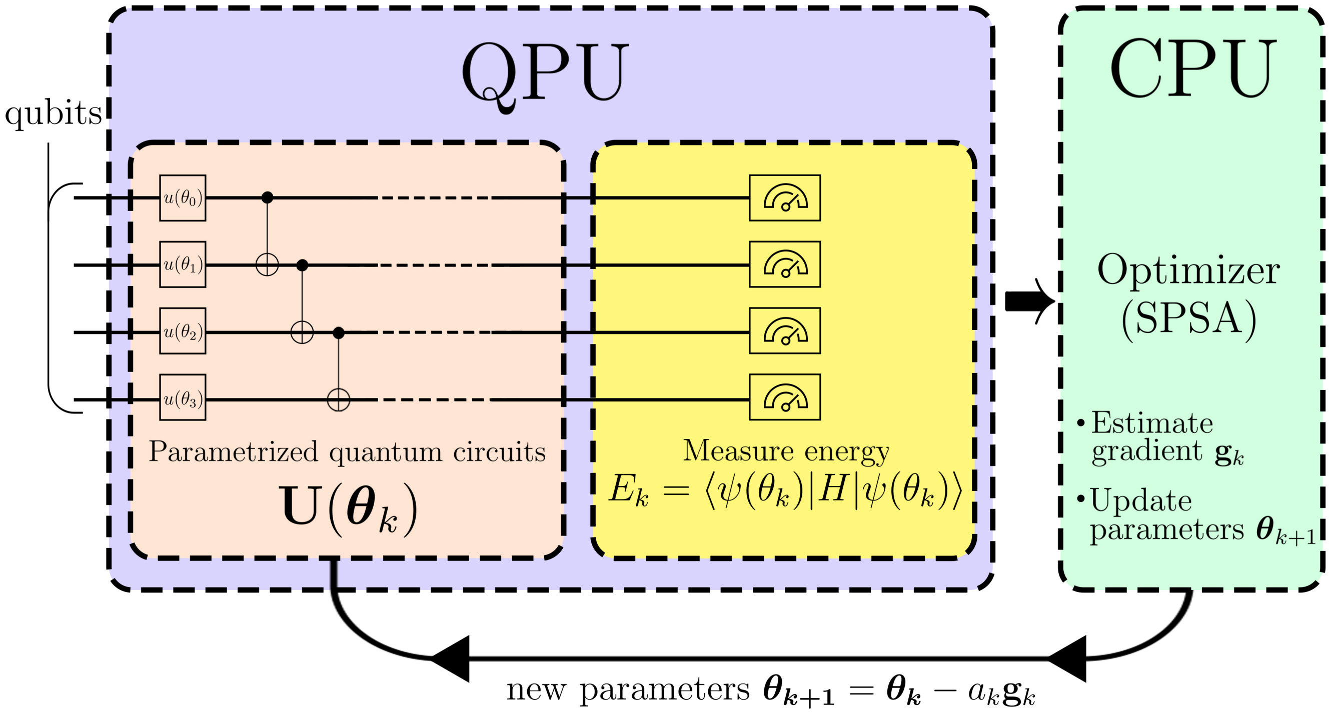

Figure S2: The iterative process and hybrid nature of the VQE. The quantum computer (QPU) is solely used for energy measurements, whereas the classical computer (CPU) is used for parameter optimization. We depict the SPSA as the optimization algorithm.

an estimation of its ground state energy on a QPU or a simulator. `Qiskit Nature`[10,11] provides an interface to the `PySCF` quantum chemistry library,[12] which is a Python toolkit that wraps C++ functions that perform Self-Consistent Field (SCF) method. Using `PySCF` through `Qiskit`, it is possible to define any molecule based on its constituent atoms and their spacial coordinates, in addition to the molecule's multiplicity or spin, as well as its charge to fix the number of electrons in the molecular system. It is also necessary to define the basis set's type and the unit of distance. These definitions are implemented by initializing a `PySCFDriver` from `Qiskit Nature`. The computations of the one- and two-body integrals for the second quantized Hamiltonian are performed internally by running the SCF algorithm to find the molecular orbitals and the Hartree-Fock reference state, then computing the electronic integrals. Running the `PySCFDriver` returns the electronic structure problem object, which contains the results of the computations mentioned above.

```python
# The BeH2 molecule
driver = PySCFDriver(
        atom="""H -1.326, 0.0, 0.0
                Be 0.0, 0.0, 0.0
                H  1.326, 0.0, 0.0
             """,
        basis='sto3g',
        charge=0,
        spin=0,
        unit=UnitsType.ANGSTROM)

# Generating the Electronic Structure Problem
molecule_problem = driver.run()
```

We are also provided with a complete active space (CAS) method to focus on a specific set of active orbitals and freeze a set of occupied orbitals, which are, in general, the core orbitals. This reduces the number of required computational resources, notably the number of necessary qubits. This is performed using the `ActiveSpaceTransformer`, which reduces the original problem to a smaller problem. The transformer is instantiated by passing in the number of active electrons and the number of active molecular (spatial) orbitals. It is worth noting that both the full and reduced Hamiltonians we obtain either from the full problem or the reduced problem include constants that are computed classically, such as the nuclear-nuclear potential and all the residue terms that result from the active space reduction. These terms must be later re-introduced in the final result, as we will see in the upcoming subsections. Below, we reduce the entire problem to only consider 2 electrons and 3 active molecular orbitals, corresponding to a total of 6 spin orbitals. These spin orbitals translate directly to qubits, first giving us 6 qubits before we reduce this number further to 4 qubits during the mapping step later.

```python
# Reducing the problem to the active space containing the 2 electrons in the 3
                                        spatial orbitals
active_space_transformer = ActiveSpaceTransformer(
                        num_electrons=2, num_spatial_orbitals=3
                        )
reduced_molecule_problem = active_space_transformer.transform(molecule_problem)
```

4.2 The Hamiltonian in terms of qubit operators

Now that we have defined and reduced the molecular problem, one must generate the Hamiltonian and translate it into operators that can be directly measured on a quantum computer (spin or Pauli operators). This transformation, corresponding to a second quantization of the Hamiltonian and a mapping, can also affect both the depth of the ansatz and the required number of measurements. In `Qiskit`, obtaining the 2nd quantized Hamiltonian is a matter of extracting it from the problem object:

```python
# Obtaining the second quantized Hamiltonian
second_q_hamiltonian = reduced_molecule_problem.second_q_ops()[0]
```

The next step is to map its ladder operators to Pauli operators. `Qiskit Nature` provides us with tools to perform this mapping, of which we will use the `ParityMapper`. The `ParityMapper` can apply a qubit tapering operation that reduces the number of qubits needed for the resulting mapped

Hamiltonian if the number of electrons in the α and β spin sectors is given. The num_particles attribute of the molecule problem object gives these two numbers. The mapped Hamiltonian qubit_op is now defined on 4 qubits, as explained above.

```
# Defining the Parity mapper
# When the number of particles is given, 2-qubit tapering is also applied
parity_mapper = ParityMapper(num_particles=reduced_molecule_problem.
                                        num_particles)
# Applying the Parity Mapping
qubit_op = parity_mapper.map(second_q_hamiltonian)
```

4.3 Ansatz circuit construction in Qiskit

The subsequent task involves selecting an ansatz that balances between computational expressiveness and practicality. It must be sufficiently expressive to approximate the ground state wave function accurately without leading to excessively deep circuits or overly complex parameterizations, making efficient training challenging. Any parameterized circuit can, in principle, be used as an ansatz, provided it acts on the same number of qubits as the mapped Hamiltonian. These can be built manually or imported from Qiskit and Qiskit Nature's circuit libraries. In our case, we shall use the anstze provided to us in those libraries. As discussed in the main text, we will be using the *UCCSD* and *Efficient SU2* anstze.

UCCSD

We remind ourselves that in the *UCCSD* ansatz, we typically evolve the Hartree-Fock initial state. We therefore must define this state for our reduced problem by passing in the number of electrons (in the α and β sectors), the number of molecular orbitals, and the used mapper to the HartreeFock constructor.

```
# Defining the Hartree-Fock initial
hf_initial_state = HartreeFock(
        num_particles=reduced_molecule_problem.num_particles,
        num_spatial_orbitals=reduced_molecule_problem.num_spatial_orbitals,
        qubit_mapper=parity_mapper
        )
```

We now build the *UCCSD* circuit by passing to UCCSD the same parameters in addition to the initial state. The initial state will thus be prepended to the *UCCSD* evolution circuit.

```
# Defining the UCCSD ansatz using the HF initial state
ansatz = UCCSD(
        reduced_molecule_problem.num_spatial_orbitals,
        reduced_molecule_problem.num_particles,
        initial_state=hf_initial_state,
        qubit_mapper=parity_mapper
        )
```

Hardware-Efficient Ansatz: Efficient SU2

*HEA*s typically do not consider the physical properties of the system at hand, and such is the case for the *Efficient SU2* ansatz. Therefore, its construction will mostly depend on the properties of the desired final quantum circuit, such as the number of qubits, the entanglement scheme, and the number of times the rotation and entanglement blocks are repeated. For a circuit with linear entanglement scheme, one repetition, and that acts on the same number of qubits as the mapped Hamiltonian, we used the definition below.

```
ansatz = EfficientSU2(
            num_qubits=qubit_op.num_qubits, entanglement='linear', reps=1
            )
```

4.4 Transpilation

In order to run the ansatz quantum circuit on a quantum computer, we must re-express it in terms of quantum gates that are natively supported by the target quantum computer. That is, decomposing the initial logical quantum gates into native physical quantum gates, as well as respecting the physical qubits' connectivity which may require reassigning qubits and re-routing two-qubit gates. This process of converting a logical circuit into a physical one is called transpilation or compilation.

To implement this in `Qiskit`, first, we define the target quantum backend, which may be a real quantum hardware or a simulator. A variety of simulators can be used in `Qiskit`'s ecosystem, ranging from several perfect simulators to simulated IBM quantum computers. In our case, we use `Qiskit Aer`'s `AerSimulator`.[10] Then, we prepare a pass manager to perform the transpilation. We set the target backend to the simulator and specify that we require no optimization in our specific case. The transpiled quantum circuit is the Instruction Set Architecture (ISA) circuit. Finally, the qubit layout of the transpiled ansatz is applied to the Hamiltonian observable, re-routing the qubits of the observable to align with those of the ansatz. This is important as we must measure the correct observables on the correct qubits.

```
# Creating a backend
# In this case, it is an SVS simulator
backend = AerSimulator()
# Creating the pass manager that transpiles the ansatz
pm = generate_preset_pass_manager(backend=backend, optimization_level=0)

# Transpiling the ansatz
# ISA stands for "Instruction Set Architecture"
isa_ansatz = pm.run(ansatz)

# Applying the layout of the ISA ansatz to the Hamiltonian observable
# This ensures that the observables qubits are the same as the ansatz qubits
isa_observables = qubit_op.apply_layout(isa_ansatz.layout)
```

4.5 Measuring eigenvalues and energies

`Qiskit` uses the *primitives* processing instructions[13] in order to interact with real quantum hardware. They are defined as the simplest building blocks of quantum applications. Two primitives are available: `Sampler` and `Estimator`. The former is used to directly measure the qubits' states, whereas the latter is used in addition to a set of observables to measure their expectation values with respect to the state defined by an input quantum circuit. For the VQE, we must use the `Estimator` primitive to measure the Hamiltonian's expectation value with respect to our parameterized ansatz.

Let us remind ourselves that the reduced Hamiltonian we have defined above omits certain constant terms that are stored in the molecule problem's instance, as mentioned in 4.1. Therefore, these constants must be added back to the expectation values that we measure using `Estimator`. At the end, given a parameterized ansatz $|\psi\rangle$, a Hamiltonian H, and a set of variational parameters $\boldsymbol{\theta}$, the sum of these omitted constants and the resulting expectation value gives us the value of the energy cost function $E(\boldsymbol{\theta}) = \langle\psi(\boldsymbol{\theta})| H |\psi(\boldsymbol{\theta})\rangle$.

The two code blocks below define the expectation value correction (interpretation) and the energy cost functions.

```
# Getting the energy value by interpreting the expectation value
# in the context of the reduced molecule problem
def interpret_exp_val(exp_val, problem):
    # Wrapping the expectation value in MinimumEigensolverResult
    sol = MinimumEigensolverResult()
    sol.eigenvalue = np.real(exp_val)
    # Interpreting the result
    return  problem.interpret(sol).total_energies[0]
```

```
# Using the Estimator primitive with the previously defined backend
estimator = EstimatorV2(mode=backend)

# Defining the energy cost function
def energy_cost_function(params):
    # Run the job and get the eigenvalue result
    estimator_job = estimator.run([(isa_ansatz, isa_observables, params)])
    estimator_exp_val = estimator_job.result()[0].data.evs
    # Return the interpreted energy value
    return interpret_exp_val(estimator_exp_val, reduced_molecule_problem)
```

4.6 The optimization

The core classical component of the VQE is the optimization algorithm. It is the classical procedure of varying a set of parameters to minimize the value of the cost function. Of the various optimizer algorithms that exist,[14–16] the Simultaneous Perturbation Stochastic Approximation (SPSA) is one of the most adequate optimizers for VQE applications, as it is designed for fluctuating cost functions,[17] and performs well under noisy conditions.[18] We will make use of SPSA to apply the Ritz' variational principle until the expectation value for the Hamiltonian is minimized. A description of SPSA's workflow is given in the main text's Optimization section.

`Qiskit`'s `SPSA` optimizer takes in the cost function to minimize as well as an initial set of parameters. During the optimization, these parameters will be varied to minimize the value of the

cost function. This will go on until a set number of iterations is reached or until a predefined termination condition is met. For our VQE, the cost function is the energy cost function, and the parameters are the ansatz parameters. We chose to limit the SPSA optimization to 250 iterations instead of setting a termination condition. This is implemented in the code below, where we also define a callback function that is run after every iteration. It stores the intermediate energy values and prints them while the VQE is ongoing.

```
# Results list to store the energy values
results = []

# The callback function runs after each iteration
def optimizer_callback(ne, params, value, step, accepted):
    global results
    results.append(value)
    print(f'Iteration {len(results):03d}  -  Energy = {value}')
```

```
# Defining the SPSA optimizer
optimizer = SPSA(maxiter=250, callback=optimizer_callback)
```

Calibration of the learning rate

The SPSA algorithm calibrates its learning rate using a first set of 50 measurements to estimate the gradient around the initial point in the search space defined by the initial parameters.[19,20] The remaining hyperparameters of SPSA are left to their Qiskit implementation's default values. (See the main text's Simulations subsection).

```
# Defining random initial parameters
initial_params = np.random.rand(ansatz.num_parameters)

# Calibrating the SPSA optimizer
learning, pert = optimizer.calibrate(energy_cost_function, initial_params)

# Setting the calibrated learning rate and perturbation series
optimizer.learning_rate = learning
optimizer.perturbation = pert
```

4.7 Running the VQE

At this point, every component of the VQE is defined and set: the molecule's Hamiltonian, the ansatz, the energy cost function, and the optimization algorithm. The VQE can then be performed by running the optimizer. The final result will contain the optimized ansatz parameters and the minimized energy value. If the optimizer reaches the global minimum of the cost function, then the minimized energy will correspond to the molecule's ground state energy.

```
# Running the VQE algorithm
# It is the SPSA algorithm that uses a quantum cost function
result = optimizer.minimize(energy_cost_function, initial_params)

# Storing the final energy and parameters
energy_result, parameters_result = result.fun, result.x
```

4.8 VQE results and post-processing

In the VQE, we are not always guaranteed to reach a good result due to the possibility of encountering local minima, barren plateaus, or simply as a result of excessive noise. In the cases where the algorithm doesn't converge towards a meaningful value, we have to restart the optimization or make any necessary changes in any of the components of the VQE's pipeline. Provided that a satisfactory result - in the limits of quantum coherent and incoherent noise - is reached, error mitigation techniques should, in general, be applied.[4,21] Several of these have been developed for the NISQ era, such as zero-noise extrapolation (ZNE), Pauli Twirling, Clifford data regression, probabilistic error amplification, and probabilistic error cancellation to name a few. In the following, we provide a brief description of ZNE, as well as a simple implementation in `Qiskit 1.2`.

4.8.1 Zero-Noise Extrapolation

ZNE[22] is an error mitigation strategy for NISQ-era quantum computing aimed at enhancing the accuracy of expectation values measurement on noisy quantum systems. The fundamental principle of ZNE involves deliberately increasing the noise levels of quantum circuits, often achieved by techniques like stretching gate durations or amplifying gate errors by repeating the gates or the whole circuit and then using the new resulting noisier data points to extrapolate back towards an estimate of what the outcome would be without noise. As an error *mitigation* technique, this approach avoids the need for additional quantum resources for complex error-correcting codes at the cost of increasing the number of quantum computations and additional classical post-processing. This makes it particularly suitable for near-term quantum devices. By mitigating the effects of noise through this extrapolation, ZNE can potentially improve the accuracy of quantum algorithms such as VQE, helping to bridge the gap between current noisy hardware and accurate quantum simulations. In the code below, we implement a simple ZNE circuits generation function that may be used with a fake QPU. The fake QPU (namely, a noisy simulator) replaces the ideal simulator we have used in 4.4. The resulting circuit will have a noise level that increases as the fold number.

```
# Since we will produce new circuits, we need another transpiler.
zne_pm = generate_preset_pass_manager(
                     optimization_level=0,
                     basis_gates=backend.configuration().basis_gates
                     )


def fold(isa_circuit, n=1):
    """Creates integer folds for a given transpiled circuit"""
    new_circuit = isa_circuit.copy()

    for _ in range((n-1)//2):
        new_circuit = new_circuit.compose(isa_circuit.compose(isa_circuit.
                                                   inverse()))

    # The new circuit must re-transpiled
    new_circuit = zne_pm.run(new_circuit)
    return new_circuit
```

Beyond error mitigation, other methods of post-processing include, for example, averaging over the last N iterations' results[20] to get a final estimate of the Hamiltonian's ground state energy.

References

(1) Hehre, W. J.; Stewart, R. F.; Pople, J. A. Self-Consistent Molecular-Orbital Methods. I. Use of Gaussian Expansions of Slater-Type Atomic Orbitals. *The Journal of Chemical Physics* **1969**, *51*, 26572664.

(2) Cramer, C. J. *Essentials of computational chemistry*, 2nd ed.; John Wiley & Sons: Chichester, England, 2004.

(3) Lewars, E. G. *Computational chemistry*, 2nd ed.; Springer: Dordrecht, Netherlands, 2010.

(4) Dobrautz, W.; Sokolov, I. O.; Liao, K.; Ros, P. L.; Rahm, M.; Alavi, A.; Tavernelli, I. Toward Real Chemical Accuracy on Current Quantum Hardware Through the Transcorrelated Method. *Journal of Chemical Theory and Computation* **2024**, *20*, 4146–4160.

(5) Szabo, A.; Ostlund, N. S. *Modern quantum chemistry*; Dover Books on Chemistry; Dover Publications: Mineola, NY, 1996.

(6) Szabo, A.; Ostlund, N. S. *Modern quantum chemistry*; Dover Books on Chemistry; Dover Publications: Mineola, NY, 1996; pp 180–189.

(7) Lewars, E. G. *Computational chemistry*, 2nd ed.; Springer: Dordrecht, Netherlands, 2010; pp 210–231.

(8) Dunning, T. H. Gaussian basis sets for use in correlated molecular calculations. I. The atoms boron through neon and hydrogen. *The Journal of Chemical Physics* **1989**, *90*, 10071023.

(9) Cohen-Tannoudji, C.; Diu, B.; Laloë, F. *Mécanique quantique - Tome III*; EDP Sciences, 2017.

(10) Javadi-Abhari, A.; Treinish, M.; Krsulich, K.; Wood, C. J.; Lishman, J.; Gacon, J.; Martiel, S.; Nation, P. D.; Bishop, L. S.; Cross, A. W.; Johnson, B. R.; Gambetta, J. M. Quantum computing with Qiskit. *arXiv* **2024**, arXiv:2405.08810.

(11) The Qiskit Nature developers and contributors Qiskit Nature 0.7.2. 2024; https://doi.org/10.5281/zenodo.7828768, Accesed: February 24th, 2025.

(12) Sun, Q. et al. Recent developments in the PySCF program package. *The Journal of Chemical Physics* **2020**, *153*, 024109.

(13) IBM Quantum. https://quantum.ibm.com/, https://quantum.ibm.com/, Accesed on: February 24th, 2025.

(14) Powell, M. J. D. An efficient method for finding the minimum of a function of several variables without calculating derivatives. *The Computer Journal* **1964**, *7*, 155–162.

(15) Powell, M. J. D. Direct search algorithms for optimization calculations. *Acta Numerica* **1998**, *7*, 287336.

(16) Sorourifar, F.; Rouabah, M. T.; Belaloui, N. E.; Louamri, M. M.; Chamaki, D.; Gustafson, E. J.; Tubman, N. M.; Paulson, J. A.; Neira, D. E. B. Towards Efficient Quantum Computation of Molecular Ground State Energies using Bayesian Optimization with Priors over Surface Topology. *arXiv* **2024**, arXiv:2407.07963.

(17) Spall, J. C. An overview of the simultaneous perturbation method for efficient optimization. *Johns Hopkins apl technical digest* **1998**, *19*, 482–492.

(18) Pellow-Jarman, A.; Sinayskiy, I.; Pillay, A.; Petruccione, F. A comparison of various classical optimizers for a variational quantum linear solver. *Quantum Information Processing* **2021**, *20*, 202.

(19) Spall, J. C. *Introduction to Stochastic Search and Optimization*; John Wiley & Sons, Ltd, 2003; pp 176–207.

(20) Kandala, A.; Mezzacapo, A.; Temme, K.; Takita, M.; Brink, M.; Chow, J. M.; Gambetta, J. M. Hardware-efficient variational quantum eigensolver for small molecules and quantum magnets. *Nature* **2017**, *549*, 242–246.

(21) Tilly, J.; Chen, H.; Cao, S.; Picozzi, D.; Setia, K.; Li, Y.; Grant, E.; Wossnig, L.; Rungger, I.; Booth, G. H.; Tennyson, J. The Variational Quantum Eigensolver: A review of methods and best practices. *Physics Reports* **2022**, *986*, 1–128.

(22) Temme, K.; Bravyi, S.; Gambetta, J. M. Error Mitigation for Short-Depth Quantum Circuits. *Phys. Rev. Lett.* **2017**, *119*, 180509.
